# Low light intensity elongates period and defers peak time of photosynthesis: a computational approach to circadian-clock-controlled photosynthesis in tomato

**DOI:** 10.1093/hr/uhad077

**Published:** 2023-04-25

**Authors:** Ting Huang, Hui Liu, Jian-Ping Tao, Jia-Qi Zhang, Tong-Min Zhao, Xi-Lin Hou, Ai-Sheng Xiong, Xiong You

**Affiliations:** College of Horticulture, Nanjing Agricultural University/State Key Laboratory of Crop Genetics and Germplasm Enhancement/Key Laboratory of Horticultural Crop Biology and Germplasm Creation in East China of Ministry of Agriculture and Rural Affairs Nanjing 210095, Jiangsu, China; College of Horticulture, Nanjing Agricultural University/State Key Laboratory of Crop Genetics and Germplasm Enhancement/Key Laboratory of Horticultural Crop Biology and Germplasm Creation in East China of Ministry of Agriculture and Rural Affairs Nanjing 210095, Jiangsu, China; College of Horticulture, Nanjing Agricultural University/State Key Laboratory of Crop Genetics and Germplasm Enhancement/Key Laboratory of Horticultural Crop Biology and Germplasm Creation in East China of Ministry of Agriculture and Rural Affairs Nanjing 210095, Jiangsu, China; The Institute of Agricultural Information, Jiangsu Province Academy of Agricultural Sciences, Nanjing 210014, Jiangsu, China; College of Horticulture, Nanjing Agricultural University/State Key Laboratory of Crop Genetics and Germplasm Enhancement/Key Laboratory of Horticultural Crop Biology and Germplasm Creation in East China of Ministry of Agriculture and Rural Affairs Nanjing 210095, Jiangsu, China; Laboratory for Genetic Improvement of High Efficiency Horticultural Crops in Jiangsu Province, Institute of Vegetable Crop, Jiangsu Province Academy of Agricultural Sciences, Nanjing 210014, Jiangsu, China; College of Horticulture, Nanjing Agricultural University/State Key Laboratory of Crop Genetics and Germplasm Enhancement/Key Laboratory of Horticultural Crop Biology and Germplasm Creation in East China of Ministry of Agriculture and Rural Affairs Nanjing 210095, Jiangsu, China; College of Horticulture, Nanjing Agricultural University/State Key Laboratory of Crop Genetics and Germplasm Enhancement/Key Laboratory of Horticultural Crop Biology and Germplasm Creation in East China of Ministry of Agriculture and Rural Affairs Nanjing 210095, Jiangsu, China; College of Sciences, Nanjing Agricultural University, Nanjing 210095, Jiangsu China

## Abstract

Photosynthesis is involved in the essential process of transforming light energy into chemical energy. Although the interaction between photosynthesis and the circadian clock has been confirmed, the mechanism of how light intensity affects photosynthesis through the circadian clock remains unclear. Here, we propose a first computational model for circadian-clock-controlled photosynthesis, which consists of the light-sensitive protein P, the core oscillator, photosynthetic genes, and parameters involved in the process of photosynthesis. The model parameters were determined by minimizing the cost function ( $\boldsymbol{\delta} =\mathbf{8.56}$), which is defined by the errors of expression levels, periods, and phases of the clock genes (*CCA1*, *PRR9*, *TOC1*, *ELF4*, *GI*, and *RVE8*). The model recapitulates the expression pattern of the core oscillator under moderate light intensity (100 μmol m ^−2^ s^−1^). Further simulation validated the dynamic behaviors of the circadian clock and photosynthetic outputs under low (62.5 μmol m^−2^ s^−1^) and normal (187.5 μmol m^−2^ s^−1^) intensities. When exposed to low light intensity, the peak times of clock and photosynthetic genes were shifted backward by 1–2 hours, the period was elongated by approximately the same length, and the photosynthetic parameters attained low values and showed delayed peak times, which confirmed our model predictions. Our study reveals a potential mechanism underlying the circadian regulation of photosynthesis by the clock under different light intensities in tomato.

## Introduction

Tomato (*Solanum lycopersicum* L.) is one of the main vegetable crops cultivated worldwide, and prefers strong light in the photosynthetic process [1]. In tomato, light intensity is an important environmental cue to conserve energy towards improvement of photosynthesis. Under low light intensity, photosynthesis is limited due to the low rates of the light-dependent reaction. As the amount of light increases, higher intensity under optimum growth conditions enhances the rate of photosynthesis in tomato [2]. Light intensity is the driving force enabling the plant to achieve a balance to conserve energy towards improvement of photosynthesis. Well-defined relationships among pigment perception and interception of light, electron transport systems, light reaction, and the carboxylation or oxygenation reactions of photosynthesis have been reported [3, 4]. Transduction from chloroplast to nucleus facilitates the highly coordinated expression of the many photosynthetic genes between these different compartments [5, 6]. Weak light limits the abundances of gene products and photosynthesis, and the absence of *Lhcb1* led to a marked reduction in production in a field experiment [7].

In order to identify the effect of light intensity on photosynthesis, experimental attempts have been conducted to control light density by managing shade avoidance [8]. Recent efforts aimed at controlling light intensity by setting diverse shade amounts and calculating light transmittances to explore photosynthetic characteristics and results suggested that increasing light transmittance enhanced the photosynthetic rate [9]. In tomato production facilities, decreased light transmittance leads to decreased light intensity, which will further affect photosynthesis, thus influencing the growth and development of tomato plants.

In the past two decades, computational approaches have been used extensively to investigate the dynamic behavior of regulatory networks related to photosynthesis [10]. Several kinetic models have been proposed to explore effective ways to enhance photosynthesis and optimize the distribution of resources to increase plant yields [11–13]. Poolman *et al*. developed the first ordinary differential equation (ODE) model to describe hysteresis in the transitions between the steady states of carbon assimilation flux induced by light intensity [14]. Ebenhöh *et al*. presented an ODE model of the dynamic regulation of eukaryotic photosynthesis to recapitulate the basic fluorescence features of short-term light acclimation [15]. In these models, photosynthesis was regulated by several pathways, including the chloroplast differentiation/de-differentiation pathway [8] and the glycolate decarboxylation pathway [10].

On the one hand, it has recently been reported that photosynthetic genes interact with the circadian clock in plants [16]. The circadian clock, an endogenous rhythm, has been repeatedly shown to be integrated to match environmental cues and regulate various physiological responses [17–20]. For example, the production of sugar from photosynthesis regulates the expression of clock genes early in the photoperiod [21]. Four pairs and two individual clock genes, *CIRCADIAN CLOCK-ASSOCIATED 1* (*CCA1*)/LHY, *PSEUDO-RESPONSEREGULATOR 9* (*PRR9*)/*PRR7*, *PRR5*/*TIMING OF CAB EXPRESSION 1* (*TOC1*), *EARLY FLOWERING 4* (*ELF4*)/*LUX ARRYTHMO* (*LUX*), *GIGANTEA* (*GI*), and *REVEILLE 8* (*RVE 8*), interlock to form the central oscillator [22–29]. The circadian periods of *CCA1*, *PRR 7*, and *TOC1* were shortened by ~4.2 hours under enhanced photosynthesis and continuous low light [30]. Photosynthesis-derived sucrose could reduce the transcriptional level of *PRR7* during the day [31]. Inhibited photosynthesis led to a phase advance of the *CCA1* rhythm by ~2 hours. The products of daytime photosynthesis cooperating with superoxide regulated the *TOC1* expression in the evening [32]. On the other hand, the circadian clock comprising transcription–translation feedback loops in turn provided circadian regulation of photosynthesis.

Photosynthesis is the process of capturing light energy and transforming it into chemical energy, and involves light harvesting, electron transport, photosynthetic carbon fixation, and ATP production [33]. These steps require masses of gene products, such as *Lhcb1* [34], *psbA* [35], *RbcL*/*RbcS* [36, 37], and *atpA* [38], which are normally repressed by *CCA1* and increase from midday to evening [39]. Different from other target photosynthetic genes, *Lhcb1* was reported to be upregulated by CCA1 [40]. Due to the fact that *GI* negatively regulates chloroplast biogenesis [41], GI may be an inhibitor of *Lhcb1*. Therefore, connecting the interactions between the central oscillator and photosynthetic genes forms an integrated photosynthetic output network. However, the kinetic effect of light intensity acting on photosynthetic output through the circadian pathway remains poorly understood.

In this work, we investigated photosynthetic efficiency through the pathway of the circadian clock network under different light intensities in tomato. We developed a first computational model where the light-induced circadian clock perceives different light intensity inputs, resulting in variations of plant endogenous rhythms and ultimately affecting the expression of photosynthesis-related genes and changes in photosynthetic parameters. We used the dynamic expressions of photosynthetic genes and the rhythmic properties of clock genes to fit the model. In addition, non-linear relationships between clock elements, photosynthetic proteins, and photosynthetic parameters were constructed to predict the effects of alternative light intensities on photosynthetic variables, which were consistent with the experiments in tomato.

## Results

### A mathematical model of the dynamics of the central oscillator captures tomato photosynthesis

**Figure 1:**
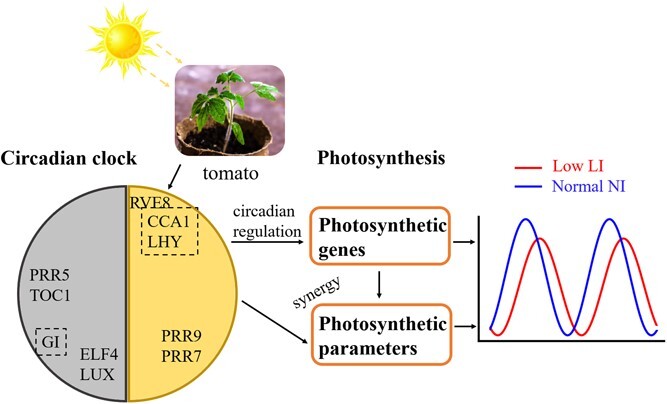
The tomato receives different light intensity inputs, leading to changing temporal evolution of photosynthetic outputs regulated by the circadian clock. The diagram conceptualizes the circadian system as a core circadian oscillator under different light cues. The photosynthetic output timings are controlled by the circadian clock, which will reset the period and phase of photosynthesis-related genes and photosynthetic parameters. The red curve and blue curve denote the time courses of variables in the model under low light intensity (low LI) and normal light intensity (normal LI), respectively.

To assess the biological significance of the circadian elements, their expression levels under constant light and mediate intensity [42] were used to fit the parameters of the core circadian oscillator built on the known regulations of these genes (Fig. 1). We used 12 differential equations (equations S1–S12 in the Supplementary Material File) that incorporated transcriptional levels and protein abundances of CL (CCA1/LHY), P97 (PRR9/PRR5), P51 (PRR5/TOC1), EL (ELF4/LUX), GI, and RVE8. The other eight equations (equations S13–S20 in the Supplementary Material File) contained active levels of related proteins or complexes, and a 21st equation represented the active proportion of light-sensitive protein (Table S1). For the schematic structure of the first four pairs of genes, we adhered to the compact circadian clock model recently described [43], which was modified from a previously model [24] including a repression rather than activation interaction between *CCA1*/*LHY* and *PRR9*/*PRR7* and a term for *CCA1*/*LHY* self-inhibition. RVE8 and PRR5/TOC1 form a negative feedback loop [44], where RVE8 is photoactivated at dawn. The dynamics of *GI* was modeled based on circadian control by CCA1/LHY, PRR9/PRR7, and PRR5/TOC1 [45].

*Lhcb1* expression (equation S22) was modeled according to CCA1 activating its transcription [40] integrated with a putative regulation incorporating GI inhibition of *Lhcb1* [41]. Lastly, the comprehensive effects of the core circadian oscillator on *Lhcb1* transcriptional activations were indicated as photosynthetic efficiency and photosynthetic parameters were represented as the clock-dependent activation of CCA1 and GI. Likewise, the transcriptional levels of other photosynthetic genes involved in photosynthesis (*psbA*, *RbcS1*, and *atpA*) were simulated by CCA1 inhibition (equations S24–S29). A full mathematical description of the model is available in the Supplementary Material File.

A typical simulated annealing algorithm (see Section 2 in the Supplementary Material File) was used to fit these different equations to the expression datasets of the tomato central oscillator under constant light and medium light intensity conditions [42]. We also used circadian properties of period and phase of genes to constrain the fit (see Materials and methods section). The fit showed that several parameters sampled randomly had a large value range. In particular, the model worked equally when excluding the post-translational regulation of *ELF3–ELF4–LUX* (EC) through interaction with COP1 [31] and GI action on ZTL stability [32]. This does not mean that these regulations do not exist or are not necessary but that they are quantitatively less relevant than others in our experimental conditions. Thus, we proceeded to apply a lower-dimensional parameter set to minimize the cost function defined by the relative error of expressions, periods, and phases of clock elements.

**Figure 2:**
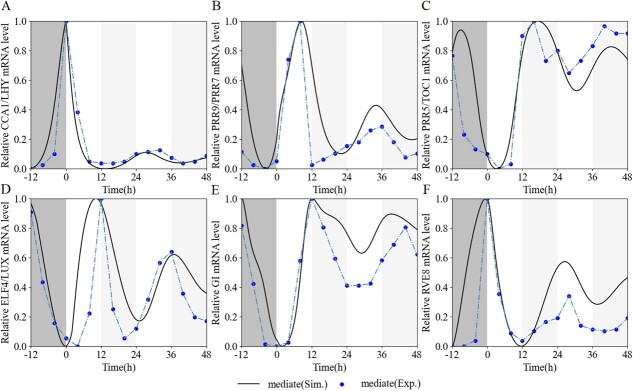
Dynamic validation of the core circadian elements under moderate light intensity. Subjective WT lines were simulated for constant light followed by five 12-hour light:12-hour dark cycles. (**A**) Expression profile of *CCA1* mRNA versus simulated *CL* level. (**B**) Expression profile of *PRR9* mRNA versus simulated *P97* level. (**C**) Expression profile of *TOC1* mRNA versus simulated *P51* level. (**D**) Expression profile of *ELF4* mRNA versus simulated *EL* level. (**E**) Expression profile of *GI* mRNA versus simulated *GI* level. (**F**) Expression profile of *RVE8* mRNA versus simulated *RVE8* level. Predictions (solid lines) of the time courses of clock elements were compared with experimental data (blue dots) extracted from Figs S1, S2, S4, and S5 in Müller *et al*. [42]. The gray and whitesmoke bands represent darkness and subjective darkness, respectively.

According to Müller *et al*. [42], the wild-type (WT) tomatoes had been previously entrained under light–dark (LD) cycles for 5 days before transferring to the constant light (LL) condition with mediate light intensity (~100 μmol m^−2^ s^−1^) (Supplementary Data Fig. S1). We redrew the data to fit our model. Furthermore, the reliability of the model was evaluated by robustness and parameter sensitivity analysis (see Section 2 in the Supplementary Material File). The robustness of the model to parameter variations was measured under 10% reductions and increments of each basic parameter. Changes of <3.8% in the period and <4.2% in the phase of CL and P51 mRNA were observed in the LL condition for simulated WT plants (Supplementary Data Fig. S2). The sensitivity of parameters was characterized by the periods and phases of the core oscillators in the LL condition. We performed simulations for quantity scanning of parameter values so that the clock genes simultaneously met the two features: the period of the clock elements were in the range of 24–28 hours, similar to that in other plants; and CL mRNA peaked in a range from 0 to 4 hours, P97 mRNA from 6 to 10 hours, P51 mRNA from 12 to 15 hours, EL mRNA from 10 to 15 hours, GI mRNA from 11 to 13 hours, and RVE8 mRNA from 0 to 3 hours after release to the LL condition. In this sensitivity analysis, each parameter ranged from 10^−3^- to $10$^3^-fold of the basal values with a logarithmic scale (Supplementary Data Fig. S3). In consequence, we obtained an optimal parameter set (Supplementary Data Table S2) producing consistent fits and predictions (Fig. 2).

### Light-flux-dependent dynamics of the tomato central oscillator

The influence of light on the pace of the plant clock is pervasive, and the regulatory dynamics of core genes are affected by light intensity [46]. To investigate the effect of light fluence rate on clock rhythms, we analyzed the rhythmic expression of the core clock genes in tomato seedlings under diverse light intensities. We used RT–qPCR for quantifying low and normal light intensities on the transcriptional levels of dominating clock elements. Having previously grown under constant moderate light flux [(MI) ~100 μmol m^−2^ s^−1^], seedling samples were collected in constant 12-hour LD cycles under different light intensities [low light intensity (LI): ~62.5 μmol m^−2^ s^−1^ and normal light intensity (NI) ~187.5 μmol m^−2^ s^−1^] (MI-to-LI and MI-to-NI; Fig. 1 and Materials and methods).

Consistent with previous reports [24, 47–49], the expressions of central clock genes showed a robust light-dependent circadian rhythm with dawn-peaking *CCA1*/*LHY* and *RVE8*, morning-peaking *PRR9*/*PRR7*, dusk-peaking *ELF4*/*LUX* and *GI*, and night-peaking *PRR5*/*TOC1* (Supplementary Data Fig. S4). Moreover, the *CCA1* and *RVE8* waveforms switched in short days to a steady-state pattern and maintained a constant low plateau excluding the hours around dawn. The peak levels of *CCA1* and *RVE8* exposure to normal light intensity were slightly less than those under low light intensity (Supplementary Data Fig. S5A and F), indicating dawn genes were expressed at weak light intensity. The same trend was observed in *PRR9* transcripts under normal and low light intensity conditions (Supplementary Data Fig. S5B), suggesting that varying light intensity within a certain range did not affect the expression peak of morning genes, whereas the transcription patterns of other genes were similar, with lower peak levels exposed to low light intensity conditions (Supplementary Data Fig. S5C–E). The relative abundances of clock genes at peak times were statistically different (*P* < .05), whereas there were no statistically significant differences in expression at other time points.

**Figure 3:**
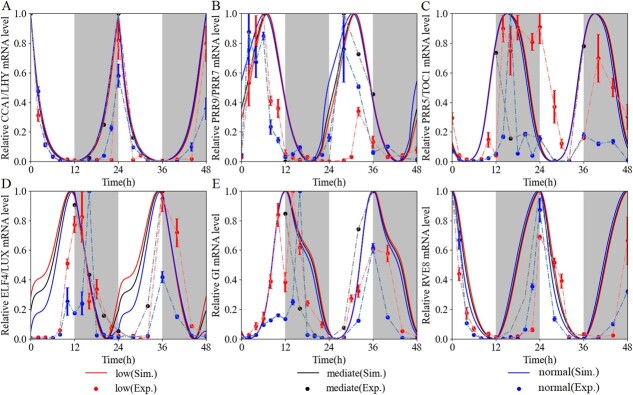
Expression patterns versus simulated temporal evolutions of the circadian clock in 12-hour light–dark cycles under different light intensities. In this experiment, WT lines were entrained in LD cycles (12 hours light/12 hours dark) under medium light intensity (~120 μmol m^−2^ s^−1^) and constant temperature (25°C) for 5 days, and then transferred to 48 hours of low and normal light intensity on day 6. (**A**) Expression profile of *CCA1* mRNA versus simulated *CL* level. (**B**) Expression profile of *PRR9* mRNA versus simulated *P97* level. (**C**) Expression profile of *TOC1* mRNA versus simulated *P51* level. (**D**) Expression profile of *ELF4* mRNA versus simulated *EL* level. (**E**) Expression profile of *GI* mRNA versus simulated *GI* level. (**F**) Expression profile of *RVE8* mRNA versus simulated *RVE8* level. The gray bands represent darkness. Solid lines indicate simulated values and dashed–dotted lines indicate experimental values. Low, medium, and normal light intensities are indicated by red, black, and blue, respectively. For (**A**–**F**), $n=3$ biologically independent samples, and the results represent mean ± standard error. All mean values are normalized to their respective maximum.

Under the MI-to-LI and MI-to-NI conditions, we simulated the expression of each core oscillator in comparison with the experimental data (Fig. 3). All values were normalized to their maximum. The simulated expression of each gene followed a pattern similar to its experimental data, except for the peak times of *EL* and *GI*, i.e. compared with the experimental data, there was a gap of ~1–2 hours on the first day, but the peak times on the second day remained consistent, possibly because the treatment was in the transition phase of light intensity variation, while the simulations had reached a stable state.

### Light-flux-specific clock entrained to light–dark cycles with different periods and phases

**Figure 4:**
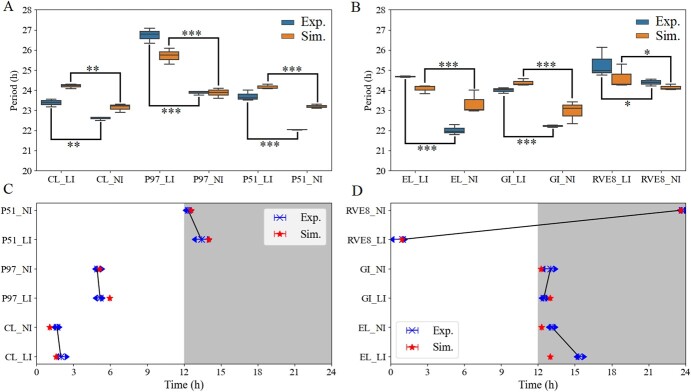
Tomato clocks show period and phase differences under different light intensities. (**A**, **B**) Periods were calculated based on experimental data and were estimated based on simulated expression profiles of the clock genes by the MFourFit method under different light intensities. Means of periods between low and normal light conditions are statistically different (*P < .05, **P < .01, ***P < .001, one-way ANOVA, Tukey *post hoc* test). Simulations and experiments under the same light intensity are not significantly different. (**C**, **D**) Peak times of clock gene expression in different light intensities under the LD condition. Plots represent the calculated peak times for the three biological repetitions. Red stars denote simulated peak times of each gene in low and normal light intensities.

The circadian clock is conserved at the transcriptional level of networks as well as core genes in plants, ensuring that biological processes are phased to the correct time of day [50]. In the expression patterns we observed phase differences between different light intensities (Figs 3 and 4, Supplementary Data Fig. S5). The low light flux peaked after the normal, and the medium was in the middle (Fig. 4B). Moreover, the emergence of period differences could be observed between low and normal fluence rates (Fig. 4A). The low intensity maintained a longer period with a range between 23.38 and 26.78 h, whereas the normal intensity ran between 22.01 and 24.38 h (Table 1). The simulations gave qualitative results that were coherent between slow and fast fluence rates (Fig. 4A, Table 1). Thus, light intensity has important effects on the period and phase of the tomato circadian clock. As light parametric entrainment followed Aschoff’s rule [51] in *Arabidopsis* [52], increasing fluence rates led to faster clock rhythms, resulting in a shorter clock period; decreasing fluence rates produced slower clock rhythms, resulting in a longer period. We verified that our results were not specific to specific genes, as we observed similar differences in periods and phases of the core clock genes (Fig. 4, Table 1). The periods and phases of experimental data were calculated by the MFourFit method (available online at https://biodare2.ed.ac.uk/documents/period-methods) with three biological repetitions. The periods and phases of simulations were estimated by three methods (MFourFit, MESA, ER Periodogram) to obtain the mean and standard deviation values.

**Table 1:** Periods and phases of the clock oscillation system induced by low and normal light intensities

	CCA1/LHY	PRR9/PRR7	PRR5/TOC1	ELF4/LUX	GI	RVE8
Phase Exp. (low)	2.01$\pm$0.24	5.13$\pm$0.14	13.43$\pm$0.42	15.39$\pm$0.17	12.49$\pm$0.08	0.75$\pm$0.19
Period Exp. (low)	23.38$\pm$0.19	26.73$\pm$0.37	23.71$\pm$0.26	24.67$\pm$0.03	23.99$\pm$0.14	25.29$\pm$0.73
Phase Exp. (normal)	1.64$\pm$0.06	5$\pm$0.15	12.34$\pm$0.07	13.14$\pm$0.11	12.94$\pm$0.33	23.76$\pm$0.09
Period Exp. (normal)	22.6$\pm$0.09	23.87$\pm$0.1	22.03$\pm$0.01	22.01$\pm$0.24	22.22$\pm 0.05$	24.37$\pm$0.16
Phase Sim. (low)	1.39	6.03	14	12.8	13.39	0.94
Period Sim. (low)	24.21$\pm$0.11	25.71$\pm$0.39	24.16$\pm$0.12	24.07$\pm$0.21	24.37$\pm 0.16$	24.62$\pm$0.58
Phase Sim. (normal)	0.97	5.13	12.56	11.79	12.84	23.59
Period Sim. (normal)	23.14$\pm$0.21	23.87$\pm 0.25$	23.19$\pm 0.1$	23.33$\pm$0.57	22.95$\pm$0.35	24.13$\pm$0.14

Exp. and Sim. indicate the phase or period calculated with experimental expression profiles and numerical simulations of the core circadian clock genes, respectively.

The model captures the core oscillator dynamics we observed experimentally (Fig. 3, Supplementary Data Fig. S5). The rhythmic periods were elongated and phases were pushed backward under low light intensity, which validated the model’s predictions.

### Clock control of the dynamics of photosynthetic genes

Light harvesting, electron transport, photosynthetic carbon fixation, and ATP synthesis are the main processes involved in photosynthesis. *Lhcb1*, *psbA*, *RbcS1*, and *atpA* are representative functional genes corresponding to these processes. In order to investigate the role of light fluence in photosynthesis, expressions of photosynthetic genes were quantified under low and normal light intensity conditions (Supplementary Data Fig. S6). The difference analysis demonstrated that under low and normal light intensities the expression was significantly different at the first daytime and at the peak time in the second LD cycle. We calculated the period and phase under MI-to-LI and MI-to-NI conditions (Table 2). Tomato plants under low light and normal light intensities ran at 24.50 $\pm$ 0.138 and 24.09 $\pm$0.08 hours, respectively. These results indicated that the lower light fluence rate was conducive to lengthening the oscillating period and to inducing a phase lag of photosynthetic genes, consistent with results for the circadian rhythm.

**Table 2:** Comparison of periods and phases between photosynthetic output model and experimental data of photosynthetic genes.

Intensity	Gene	Period (hours; mean $\pm$s.d.)	Phase (hours; mean$\kern0.5em \pm$s.d.)
LI (Exp.)	*Lhcb1*	24.68$\pm$0.28a	6.39$\pm$0.3a
NI (Exp.)		24$\pm$0.32b	5.76$\pm$0.28b
LI (Sim.)		24.23$\pm$0.03ab	6.71$\pm 0.14$a
NI (Sim.)		24.16$\pm$0.12b	5.22$\pm 0.29$b
LI (Exp.)	*psbA*	24.65$\pm$0.18a	12.43$\pm$0.32a
NI (Exp.)		24.19$\pm$0.22ab	11.78$\pm$0.19ab
LI (Sim.)		24.44$\pm$0.12a	12.16$\pm$0.03a
NI (Sim.)		23.98$\pm$0.07b	10.91$\pm$0.07b
LI (Exp.)	*RbcS1*	24.54$\pm$0.26a	21.02$\pm$0.30a
NI (Exp.)		24.13$\pm$0.23ab	19.68$\pm$0.51b
LI (Sim.)		24.48$\pm$0.14a	20.62$\pm$0.12a
NI (Sim.)		24.19$\pm$0.15ab	19.85$\pm$0.11b
LI (Exp.)	*atpA*	24.50$\pm$0.16a	21.66$\pm$0.18a
NI (Exp.)		24.04$\pm$0.11b	20.13$\pm$0.09ab
LI (Sim.)		24.54$\pm$0.25a	20.97$\pm$0.05a
NI (Sim.)		24.06$\pm$0.13b	20.19$\pm$0.09ab

Significant differences are indicated $P<.05$ by letter a and b based on Duncan’s multiple range test under low and normal light intensities. LI and NI denote the genotypes under low light intensity and normal light intensity. Exp. and Sim. indicate the phase or period calculated with experimental expression profiles and numerical simulations of photosynthetic genes, respectively.

*Lhcb1* function is essential for tomato photosynthesis. CCA1 has been identified as a positive regulator of *Lhcb1* in plants [33, 34, 53, 54]. We first tested a simple photosynthetic output model where *Lhcb1* mRNA was only activated by CL (equation S22b in the Supplementary Material File). We simulated the temporal evolution dependent on the parameters ${v}_7$, ${k}_7$, and ${K}_{17}$ achieved by trial and error (Supplementary Data Fig. S7A). The simulation results showed qualitative variations in phase and period, but the estimated phase of simulations was significantly different from the calculated values of experimental data. Moreover, the half-width of the peak in *Lhcb1* expression was relatively wide, which led to a level inconsistent with the experimental results of low expression at night. We suspected that there was another promoter of *Lhcb1* in the circadian network.

**Figure 5:**
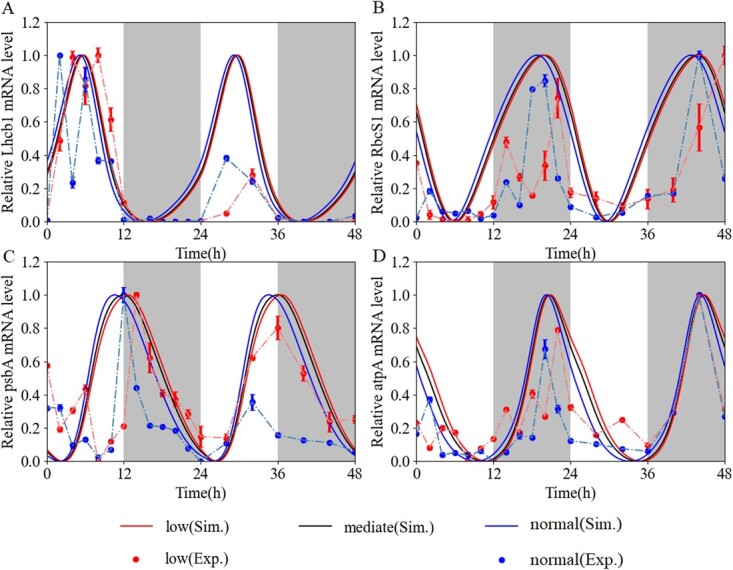
Dynamic behaviors of photosynthetic genes controlled by circadian clocks in WT. Simulated expression of *Lhcb1* (**A**), *RcbS1* (**B**), *psbA* (**C**), and *atpA* (**D**). Red dots and blue dots are corresponding expression profiles under low light and normal light intensities, respectively. The number of biological replicates is 3. Values are normalized to their respective Min–Max scaling. The gray bands denote dark, while the white bands represent light.

*GI* was reported to negatively regulate chloroplast biogenesis [35]. As the chloroplast is essential for photosynthesis [55], we subsequently extended a computational model in which *Lhcb1* was synergistically regulated by activator CL and inhibitor GI (equation S22 in the Supplementary Material File). Compared with the control model, our model has one more parameter, ${K}_{18}$. The simulated expression pattern (Fig. 5A, Supplementary Data Fig.S7B) suggested that our model had a better fitness than the controlled one (Supplementary Data Fig. S6A). We calculated the sum of squares error (SSE) between simulated values with relative experimental data. The SEEs of our model under low and normal light intensities were 1.28 and 1.52, respectively, whereas they were 4.66 and 5.30 in the control model, both within standardized data sets. In addition, the period and phase of *Lhcb1* abundance were not significantly different from those in experimental expression.

### Photosynthetic light-response and CO_2_-response curves

**Figure 6:**
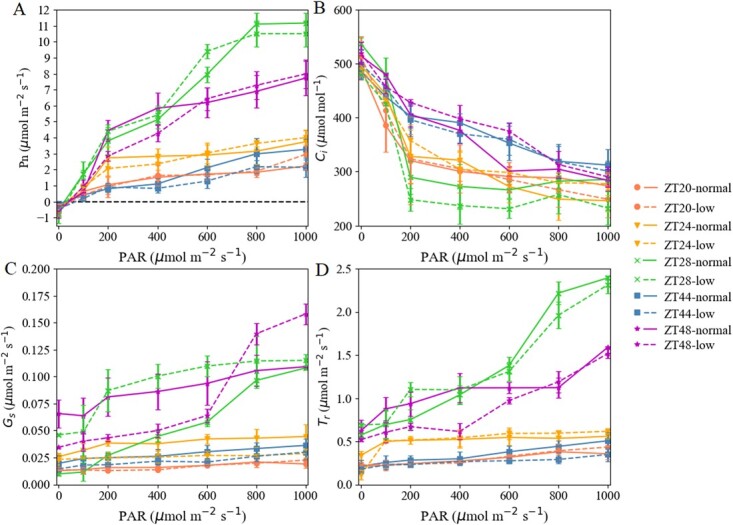
Dynamic photosynthetic light-response curves of photosynthetic parameters. ${P}_{\mathrm{n}}$, net photosynthetic rate; ${C}_{\mathrm{i}}$, intercellular CO_2_ concentration; ${G}_{\mathrm{s}}$, stomatal conductance; ${T}_{\mathrm{r}}$, transpiration rate at seedling stage of tomato under different light intensities around dawn (measurements were made at a CO_2_ concentration of 500 μl l^−1^). ZT indicates zeitgeber time, the time point of light intensity treatment. Normal intensity, 185 μmol m^−*2*^ s^−1^; low intensity, 65 μmol m^−*2*^ s^−1^. Vertical bars represent mean ± standard deviation (*n* = 20).

**Figure 7:**
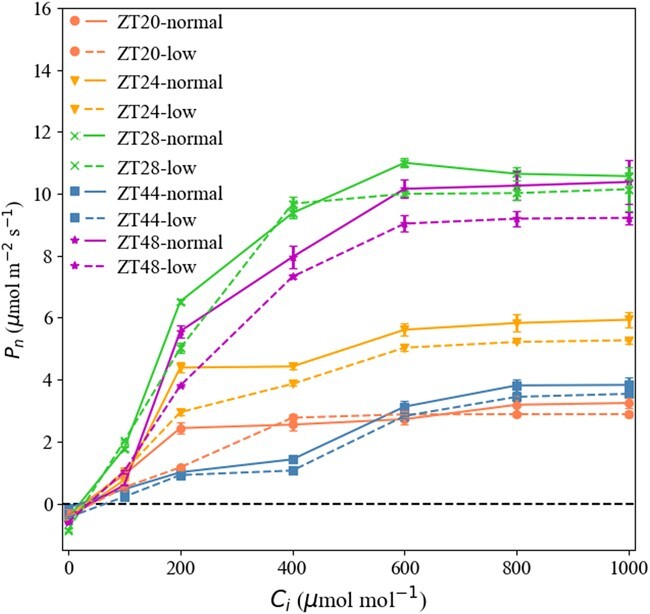
Dynamic photosynthetic CO_2_-response curves of tomato seedlings under different light intensities around dawn (measurements were made at a PAR of 600 μmol m^−2^ s^−1^). Vertical bars represent mean ± standard error (*n* = 20).

The light-response and CO_2_-response curves of tomato leaves under different light intensities around dawn are presented in Figs 6 and 7. These curves indicate dynamic variations of ${P}_n$ (net photosynthetic rate), ${C}_i$ (intercellular CO_2_ concentration), ${G}_s$ (stomatal conductance), and${T}_r$ (transpiration rate) as photosynthetically active radiation (PAR) during the seedling stage of tomato. Photosynthetic parameters in the light intensity mode had no significant difference ($P>.05$) compared with those in the zeitgeber time (ZT) mode, which were examined across dawn. Higher ${P}_n$ (66.7 and 49.6%) and ${T}_r$ (52.4 and 48.6%) were recorded at ZT28 and ZT48, respectively (Fig. 6A and 6D). Likewise, ${P}_n$ and ${T}_r$curves became constant with increasing PAR, except at ZT28 and ZT48. Treatments with the highest to lowest ${P}_n$ and ${T}_r$ under different PAR levels (>400 μmol m^−2^ s^−1^) were in the following order: ZT28 > ZT48 > ZT24 > ZT44 > ZT20.

For ${G}_s$, the values under the normal light intensity had significant (*P* $<.05$) discrepancy compared with those under low light intensity among ZT24, ZT28, and ZT48 (Fig. 6C). At ZT28 and ZT48, ${G}_s$ curves showed higher (35.3 and 36.5%) values. However, different from ${P}_n$ and ${T}_r$ curves, ${G}_s$ values under low light intensity exceeded those under high light intensity. Otherwise, there were no significant (*P*$>.05$) increases in ${C}_i$ curves in a declining manner (Fig. 6B). Similarly, the smaller dynamic changes of ${G}_s$ and ${C}_i$ values occurred with the higher PAR levels. The light-response results might show that daybreak is an important time for the clock and for relaxation of photosystems. This may be controlled by the dawn-phased circadian clock CCA1/LHY.

To simulate the CO_2_-response curve, we used the package plantecophys of software R. Under both light intensity modes and different ZTs, the CO_2_-response curves indicated that ${P}_n$ increased significantly in ${C}_i$ (Fig. 7). ${P}_n$ values under both the normal and low light intensities at ZT28 and ZT48 were significantly ($P<.05$) higher (44.5%) than levels at ZT24, and 4- to 5-fold the values at ZT 20 and ZT44. Likewise, higher light-intensity treatments produced higher accumulations of ${P}_n$. The curve was close to saturation when ${C}_i$ was >600 μmol mol^−1^.

### Circadian rhythm variations of photosynthetic parameters in tomato leaves

To characterize the photosynthetic efficiency (${P}_i$), we modelled the photosynthetic parameters in terms of CCA1, GI, Lhcb1, psbA, RbcS1, and atpA. The dynamic equations are defined by

$$ {P}_{\mathrm{i}}={\alpha}_{\mathrm{i}}+\left(\frac{{\left[\mathrm{CL}\right]}^2}{K_{i1}^2+{\left[\mathrm{CL}\right]}^2}+\sum_j\frac{{\left[{X}_j\right]}^2}{H_{ij}^2+{\left[{X}_j\right]}^2}\right)\frac{K_{i3}^2}{K_{i2}^2+{\left[\mathrm{GI}\right]}^2} $$

where $\left[\mathrm{CL}\right]$ and $\left[\mathrm{GI}\right]$ represent the abundance of CCA1 and GI, $\left[{X}_j\right]$ ($j=$ 1, 2, 3, 4) denote the protein levels of Lhcb1, psbA, RbcS1, and atpA. The other parameters have the following meanings: $i$ denotes the four photosynthetic parameters ${P}_{\mathrm{n}}$, ${G}_{\mathrm{s}}$, ${C}_{\mathrm{i}}$, and ${T}_{\mathrm{r}}$; $\mathrm{\alpha}$ is the basic photosynthetic rate; for a given photosynthetic parameter $i$, ${K}_{i1}$, ${K}_{i2}$, ${H}_{ij}$ are the intensities of CCA1 activation, GI inhibition, and Lhcb1, psbA, RbcS1, atpA promotion on ${P}_{\mathrm{i}}$, respectively. These kinetic parameters differ in different photosynthesis rate models and have variant values under normal light and low light conditions as well.

**Figure 8:**
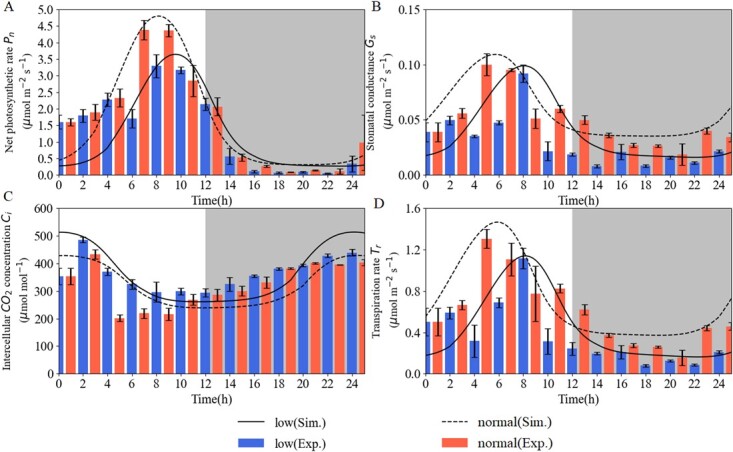
Circadian rhythm variation of photosynthetic parameters in tomato leaves. During the 24-hour photoperiod, net photosynthetic rate (${P}_{\mathrm{n}}$), stomatal conductance (${G}_{\mathrm{s}}$), intercellular CO_2_ concentration (${C}_{\mathrm{i}}$) and transpiration rate (${T}_r$) are shown in panels **A**, **B**, **C**, and **D**, respectively. Photosynthetic parameters were modeled by Hill kinetics (non-linear) on the accumulations of CCA1, GI, and photosynthetic genes. The black solid and dashed lines denote photosynthetic simulations. The histograms indicate mean values $\pm$ standard errors with three biological replicates.

As shown in Fig. 8, ${\boldsymbol{P}}_{\mathbf{n}}$ showed a higher peak under normal light prior to that under the low light condition, with the peak appearing at 6 and at 8 hours, respectively. ${\boldsymbol{P}}_{\mathbf{n}}$ was small at 14–24 hours with an evening depression. ${\boldsymbol{G}}_{\mathbf{s}}$ rose significantly at 4 hours under normal light but at 8 hours under low light. Additionally, ${\boldsymbol{G}}_{\mathbf{s}}$ tended to be flat and low at 14–22 hours under low light, and remained high at 2–14 hours under normal light. The diurnal variation of ${\boldsymbol{C}}_{\mathbf{i}}$ was in the lowest state at 4 hours and sharply increased at 8–12 hours under normal light. Interestingly, the increasing peak at 2 hours under normal light was consistent with a lift under low light, followed by a linear increase in the evening. The diurnal variations of ${\boldsymbol{T}}_{\mathbf{r}}$ under low light or normal light intensity had a similar pattern to that of ${\boldsymbol{G}}_{\mathbf{s}}$ variations. ${\boldsymbol{T}}_{\mathbf{r}}$ experienced a decline, rise, decline, and slow rise at 0–4, 4–8, 8–14, and 14–16 h, respectively, showing a W shape under low light. Inversely, ${\boldsymbol{T}}_{\mathbf{r}}$ experienced an increase, decrease, slight increase, and decrease at 0–4, 4–8, 8–10, and 10–20 h, respectively, showing an M shape under normal light. Figure 8 also shows that the fitted functions worked well (black dashed lines and full lines). The simulations suggested that low light intensity could cut down the accumulation and produce a retreat in the peak time of photosynthetic parameters (Fig. 8A, B, and D). This is consistent with the phenomenon of central oscillators and photosynthetic genes.

## Discussion

Based on the free-running expression data [42], we developed a computational model for the circadian clock system in tomato. The core circadian clock model in tomatoes was transplanted from the model of *Arabidopsis thaliana* [43], which was extended simultaneously based on the compact model of four gene pairs in [24] and the complex 10-variable model in [28]. Compared with the De Caluwé model [24] and the Pokhilko model [28], a significant improvement is the introduction of a positive feedback loop into the core system. We recast *RVE8* and *GI* genes into the previous network only comprising negative feedback loops [56]. The mutative genotypes, phenotypes, and perturbed rhythms of the core clock elements in *rve8* mutant and RVE8-OX suggested that *RVE8* is necessary for the core clock [43]. GI protein remained stable together with ZTL in a light-dependent manner [28]. The EC was reported to be necessary for the rhythmicity of the *lhy*/*cca1* mutant [57]. Moreover, it has been identified that the circadian MYB-like transcription factor RVE8 interacts with its transcriptional coactivators LNK1 and LNK2 to promote the expression of evening-phased clock genes [58]. Thus, the novel circadian networks constructed in this paper contain several protein complexes: the triple ELF3–ELF4–LUX protein complex (EC), the RVE8-LNK1 protein complex (RL), and the ZTL-GI protein complex (ZG), together with post-translational regulation of clock proteins [30, 44, 48, 59–61].

The circadian clock was entrained by LD cycles and light intensity with the constant growth and treatment temperature (25°C). Light transfers energy to the circadian clock via the photosensitive protein P, facilitating the transcription and translation of clock elements [56]. In addition, CONSTITUTIVE PHOTOMORPHOGENIC 1 (E3 ubiquitin ligase COP1) acted as a light receptor regulating EC, which has been reported as a key repressor of photomorphogenesis [62]. COP1 had a similar mechanism to protein P, with light-induced degradation and dark-induced accumulation [63]. Due to high conservation, light-dependence, and shuttling between the nucleus and cytoplasm [64], we presented three protein variables: COP1c (cytoplasmic COP1), COP1n and COP1d (nuclear COP1 in night and day) [28] in our light signal pathway. Interestingly, recent analyses of gene expression controlled by light signaling pathways have clearly placed ELONGATED HYPOCOTYL 5 (HY5) at one of the transcriptional network centers [65, 66]. HY5 can regulate a train of genes involved in promoting the transcription of the circadian clock [67]. A famous partner of HY5 is COP1 [68]. Suppression of COP1 activity is conducive to transducing the signals to HY5 [69] and mediating HY5 degradation in the dark [70].

**Figure 9:**
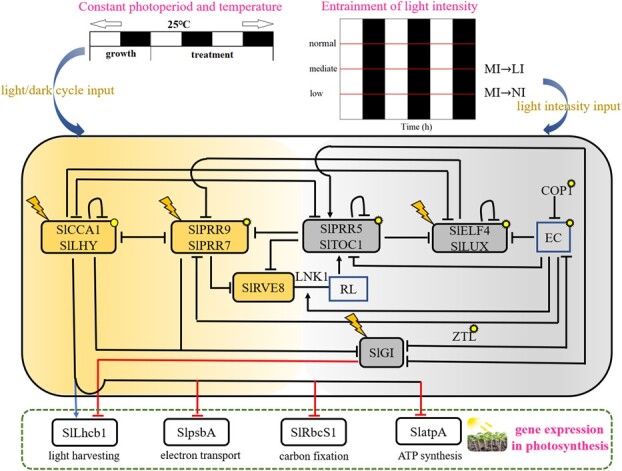
Schematic tomato circadian clock for photosynthetic output. All pairs of genes have similar expression profiles, regulators, targets, and defects in WT and loss-of-function mutant lines. Solid lines with blunt ends indicate genes functioning as repressors in the negative feedback loops. Arrows indicate genes acting as activators in the regulatory network. The red line denotes putative regulation based on research [41]. Rectangles with curved angles denote gene variables including mRNA and protein. Blue rectangles indicate protein complexes. Lightning bolt symbols represent light activation of transcription of specific genes. The small yellow smooth circles and jogged circles represent light-dependent degradation of mRNA and protein, respectively.

After rebuilding the morning loop with *RVE8* and evening loop with *GI*, we connected them to the previous main clock circuit and re-examined the correct regulation by testing the free-running rhythms of the clock. For the sake of simplicity, these main clock genes are integrated into a core circadian oscillator (Fig. 9). We used mathematical modeling to demonstrate a good correspondence of the new model to a wide spectrum of experimental data, while, compared with our model, the photosynthetic genes controlled by the core clock by the De Caluwé model [24] had bad fitness, both in waveforms and circadian features (Supplementary Data Fig. S8). The biggest drawback of this control model was that different light intensities had no effect on the period and phase of photosynthetic genes.

Generally, light intensity depends on transitions of photons [71]. A Poisson chance model was proposed to describe the reaction rate proportional to light intensity [72]. For simplicity, the three light intensities during the day functioned as fixed proportional values. The effect of different light intensities on the transcription of clock was modeled by the function $\frac{\boldsymbol{I}}{{\boldsymbol{I}}_{\boldsymbol{A}}\times{\boldsymbol{I}}^{\mathbf{2}}+{\boldsymbol{I}}_{\boldsymbol{B}}\times \boldsymbol{I}+{\boldsymbol{I}}_{\boldsymbol{C}}}$, where $\boldsymbol{I}$ denotes light intensity and ${\boldsymbol{I}}_{\boldsymbol{A}}$, ${\boldsymbol{I}}_{\boldsymbol{B}}$, and ${\boldsymbol{I}}_{\boldsymbol{C}}$ are constant. The light intensity function interacted with protein P in a light-dependent manner. If ${\boldsymbol{I}}_{\boldsymbol{A}}=\mathbf{0}$, ${\boldsymbol{I}}_{\boldsymbol{B}}=\mathbf{1}$, ${\boldsymbol{I}}_{\boldsymbol{C}}=\mathbf{0}$, the system is under mediate light intensity; $\frac{\boldsymbol{I}}{{\boldsymbol{I}}_{\boldsymbol{A}}\times{\boldsymbol{I}}^{\mathbf{2}}+{\boldsymbol{I}}_{\boldsymbol{B}}\times \boldsymbol{I}+{\boldsymbol{I}}_{\boldsymbol{C}}}>\mathbf{1}$ ($\frac{\boldsymbol{I}}{{\boldsymbol{I}}_{\boldsymbol{A}}\times{\boldsymbol{I}}^{\mathbf{2}}+{\boldsymbol{I}}_{\boldsymbol{B}}\times \boldsymbol{I}+{\boldsymbol{I}}_{\boldsymbol{C}}}$) indicates that the system is under high (low) light intensity. Our model is capable of reproducing periods and phases under the free-running condition and LD cycles. In particular, the model follows Aschoff’s rule [51], as increasing light fluence rates lead to faster speeds of the clock.

The core circadian oscillator was confined to simulation of hypocotyl growth [73] and cold stress response [74], whereas photosynthetic metabolism is also an important agronomic trait. The circadian rhythm is endogenous and self-sustaining; it drives temporal gene expression and affects the diurnal patterns of photosynthesis [75]. Through genetic, molecular, physiological, biochemical, and functional genomics analysis, significant developments have been made in identifying genes and molecular mechanisms underlying the relationship of light intensity and photosynthesis [76]. The photosynthetic genes associated with photosystem II are generally used to characterize photosynthesis. Biological experiments can have the characteristics of long duration and high consumption of labor and financial resources. With mathematical modeling it is easy to compare the dynamical differences among photosynthetic genes across different low light intensities. The transcription factor CCA1 was originally isolated as a protein binding to an *Lhcb* promoter to activate the corresponding expression [40]. Undoubtedly, CCA1 was a potential master regulator of *Lhcb1* among the circadian oscillators.

The regulated mechanism of the main genes of the photosynthetic process involving other clock elements seems not to be clear. Our photosynthesis-response model (equation 22 in the Supplementary Material File) represents the *Lhcb1* gene as being upregulated by CCA1 and downregulated by GI. The putative regulation was based on results from Cha *et al*. [41], which provide evidence for a genetic link between GI and chloroplast biogenesis. The numerical simulation and quantitative analysis demonstrated that our model was consistent with the experimental results qualitatively and quantitatively. The control model whereby CCA1 was taken to be the only transcription factor of *Lhcb1* could capture the experimental data qualitatively [30], but the simulated period and phase were significantly different from the experimental results. This indicated that the other clock was critical for capturing the observed experimental photosynthetic behaviors under different light intensities [77]. It will be interesting in future studies to validate the putative interaction whereby GI is a candidate transcription factor of *Lhcb1* and to investigate the underlying molecular mechanisms. Meanwhile, lower light intensity was required for the robust circadian rhythm of *Lhcb1* to be maintain [78] under the LL condition. When the light fluence rate exceeded a certain threshold, the rhythm of *Lhcb1* was absent and remained at a high level. Based on Hopf bifurcation analysis, the rhythm of *Lhcb1* under the LL condition theoretically depended on the value of the parameters.

The quantitative mismatch of the control model might be attributed to hidden mechanisms or the absence of some important genes in the light response pathway. Andronis *et al*. [53] found that the above-mentioned bZIP transcription factor HY5 could interact with CCA1 to regulate *Lhcb1* circadian expression. In the *hy5* mutant, *Lhcb1* ran fast*.* Moreover, HY5 specifically bound the G-box element of the *Lhcb1* promoter. On the other hand, photosynthesis has a marked effect on the entrainment and maintenance of robust circadian rhythms to regulate circadian clock[16, 30].

In the output of photosynthesis, gas exchange, stomatal conductance, and CO_2_ assimilation are under circadian clock control. Clock element *ZTL* transcription in WT is essential for the rhythmic patterns of CO_2_ fixation and stomatal conductance under LD cycles [79]. Circadian rhythms has been reported to account for 15–25% and 30–35% of daytime oscillations in photosynthesis and stomatal conductance, respectively, across C3 and C4 plants [75]. Circadian oscillations in photosynthesis and stomatal conductance response under constant light can be accessed potentially as a driver of diurnal gas exchange [80, 81]. Since GI can induce the transcription of *CCA1* and *LHY* and interact with the F-box protein ZTL in a light-enhanced manner [24], we speculated that CCA1 and GI directly regulate the accumulation of gas exchange as well as indirect control by ZTL.

In addition to utilizing genes to characterize photosynthesis regulated by the circadian clock, physiological models and state models have been presented to describe photosynthetic biomass growth. Physiological models aim to describe the dynamic behavior of photosynthetic growth by approximating the actual mechanisms [82]. State models are instead based on the photosynthetic unit (PSU), which consists of the light-harvesting complex, the reaction center, and the associated apparatus [83]. These models are instrumental for optimizing industrial cultivation systems [84].

The model prediction of photosynthesis is valuable for crop yields based on a certain expected set of light intensity and photoperiod combinations, ultimately leading to drastic cuts in experimental time and resources. As the updated model is fairly accurate, the modeling approach leads to the possibility of developing similar models for exposure to other environmental cues, such as light quality, temperature, and water, and for other physiological outputs, such as stress tolerance [85–88].

## Conclusions

Genes subject to circadian clock regulation are central to many important physiological processes. Currently available models of the plant circadian clock are limited in their applications due to lack of knowledge about photosynthesis metabolism. The expressions of genes involved in photosynthesis have specific features that reflect comparative characteristics of light intensity changes. It is meaningful to model these uncertain processes at the molecular level. This paper mainly aimed to construct a schematic structure of photosynthesis, sought a set of optimal parameters, made model reliability analysis, and introduced the light intensity function to the circadian clock. Then we analyzed the expression profiles of photosynthesis-related genes regulated by the clock elements and developed a computational model for tomato photosynthesis through the circadian pathway. Numerical predictions and experimental data were compared and the abundances of photosynthetic parameters were fitted by non-linear functions of the clock elements and photosynthetic genes. This paper is mainly concerned with the establishment of the model under different intensities, neglecting other environmental cues, such as light quality, the light–dark cycle, and temperature. These factors can be taken into account in future research.

## Materials and methods

### Plant materials, growth conditions, and light intensity treatment

All experiments were performed at Nanjing Agricultural University (188.84° E, 32.04° N). Tomato seeds from the popular variety ‘He Zuo 906’ were sown in plastic pots containing a soil/vermiculite/perlite mixture (2:2:1) in a growth chamber with 12 hours light at 25°C/12 hours dark at 22°C and 70–80% relative humidity. After 2 months, healthy tomato plants that reached the five-leaf stage were first transferred into growth chamber with constant light intensity of ~ 100 μmol m^−2^ s^−1^ (MI) and then transplanted into two controlled-environment growth chambers with light intensity of ~62.5 (MI-to-LI) and ~ 187.5 μmol m^−2^ s^−1^ (MI-to-NI), respectively, a distance of 20- to 30-cm between the LED panel and the plants, and a day–night photoperiod of 12:12 hours and constant 25°C. Plants were adjusted daily to ensure well-rounded lighting and watered daily early and late in the day (Supplementary Data Fig. S1). Tomato leaf samples were harvested every 2 and 4 hours during the separate first and second light/dark cycles with 0 hours as control. All samples were immediately frozen in liquid nitrogen and then stored at −80°C. Organs from three individual plants were pooled and ground prior to RNA extraction.

### Data collection

The data (relative transcriptional levels of the core circadian elements at the light intensity 100 μmol m^−2^ s^−1^) for model parameter estimation were extracted from the published paper of Müller *et al*. [42] by using the software ImageJ (https://imagej.nih.gov/ij/). The data were redrawn simultaneously through the online website (https://apps.automeris.io/wpd/index.zh_CN.html) to reduce manual errors and the standard deviation of retrieved data was set to <.05. The specific implementation steps were as follows. In the first step, we downloaded the required expression chart. In the second step, the expression data were directly extracted and transformed into relative expressions. The detailed procedure can be found in the supplementary material of Zhang *et al*. [89]. In the third step, according to the numerical simulation results of the model, the extracted relative expressions were scaled in a reasonable range, such as data normalization.

### Model description and construction

Based on the schematic structure (Fig. 9), the model contains 29 variables (Supplementary Data Table S1) and 100 parameters (Supplementary Data Table S2), whose values were obtained through minimizing a cost function to qualitative dynamics. Following the compact model proposed by De Caluwé *et al*. [24], we appended the important clock genes *GI* and *RVE8* to the main clock, forming a circadian network of eight gene pairs. Equations (S1)–(S20) (see Supplementary Material File) describe the temporal expression profiles of the clock gene variables (including several complexes), where MCL, MP97, MP51, MEL, MGI, and MRVE8 denote the core mRNAs listed in the structure network (Fig. 9) and CL, P97, P51, EL, GI, and RVE8 represent the concentrations of the corresponding proteins. The light perception of the core circadian oscillator was modeled by the light-sensitive protein P (equation S21), which accumulated at night and degraded during the day [90]. Photosynthesis as a metabolic output was controlled by the circadian clock mainly via transcription. In the dynamic modeling, the Hill function and the mass action law were used for transcription, translation, and degradation reactions. Light and darkness are represented by the parameters $\mathrm{L}$ and $\mathrm{D}$, respectively. The value 1 denotes lights on, 0 otherwise. The light intensity was divided into three gradients at normal (~187.5 μmol m^−2^ s^−1^), mediate (~100 μmol m^−2^ s^−1^) and low (~62.5 μmol m^−2^ s^−1^) levels under constant temperature (25°C). Equations (S22–S29) denoted the time course of photosynthesis-related genes and protein regulated simultaneously by circadian elements (CCA1 or GI). Model robustness (Supplementary Data Fig. S2), parameter sensitivity (Supplementary Data Fig. S3) and Hopf bifurcation (Supplementary Data Fig. S4) were analyzed to determine the reliability of the model.

### Total RNA extraction and gene expression analysis by RT–qPCR

A Plant Total RNA Isolation Kit (Proteinssci, Shanghai, China) was applied to extract the total RNA from tomato leaf samples. The successfully extracted RNA samples were then reverse-transcribed into cDNA using HiScript II QRT SuperMix (Yeasen, Nanjing, China). The RT–qPCR was conducted according to the manufacturer’s protocol on a Bio-Rad CFX96 real-time PCR platform. The total volume of each reaction was 20 μl. The PCR conditions used were as follows: 95°C for 5 minutes, 40 cycles at 95°C for 10 seconds, 60°C for 30 seconds, and melting curve analysis (65–95°C, increasing 0.5°C every 5 seconds). The tubulin gene (*Solyc04g077020*) was used as a reference gene for data normalization. The relative gene expression levels were calculated using the 2^−ΔΔCT^ method. Three biological replicates were conducted to calculate the mean ± standard error values. All of the primer sets used in this study are listed in Supplementary Data Table S3.

### Determination of photosynthetic parameters

The photosynthetic parameter variations of tomato leaves were measured by a portable photosynthetic apparatus (LI-6400XT, LI-COR, USA). Gas exchange, including net photosynthetic rate (${P}_{\mathrm{n}}$), stomatal conductance (${G}_{\mathrm{s}}$), intercellular CO_2_ concentration (${C}_{\mathrm{i}}$), and transpiration rate (${T}_{\mathrm{r}}$), was measured. Measurement was conducted following a standard procedure as well as appropriate modifications when required [56]. The second leaf from the top of the tomato plant was selected for this measurement. The leaf area of the standard measuring head was 1.3 cm^2^ under atmospheric CO_2_ concentrations (~400 μmol mol^−1^). After dark adaptation for 30 minutes, the fluorescence induction kinetics were measured. Then, the light response curves (LRCs) were recorded immediately under the light intensity gradient of 0, 100, 200, 400, 600, 800, 1000 μmol m^−2^ s^−1^. The duration of each light intensity was 30 seconds and the saturation pulse was 30 000 μmol m^−2^ s^−1^ for 300 ms. All measurements were conducted at room temperature (25 ± 2°C) with 70–80% relative humidity. The light intensity for photosynthetic gas exchange measurement was 200 μmol m^−2^ s^−1^ with 70–80% relative humidity; leaf chamber temperature was 25°C and CO_2_ concentration 400 μmol m^−2^ s^−1^. Data presented are the average of three biological repeats ± standard error.

### Numerical simulation and statistical analysis

We simulated our ODE model utilizing Python 3.10.4, which is freely available (https://www.python.org/downloads/). In Python the classical fourth-order Runge–Kutta method was used to obtain the numerical solutions of ODEs (see Section 3 in the Supplementary Material File). The experimental free-running expression data of clock elements under mediate light intensity were extracted from Müller *et al*. [42]. All samples were assessed independently at least three times, and all data are presented as the mean ± standard error. The statistical difference analysis was performed with the Rstudio 3.6.0 (https://www.rstudio.com/products/rstudio/download/). Independent *t*-tests and one-way ANOVA were used to analyze the data, and Duncan’s multiple comparisons were used for sample comparisons at a significance level of ^*^P < .05.

## Supplementary Material

Web_Material_uhad077

## Data Availability

Data are available in figures of the article and its supplementary materials.
