# Supplementary material for: Low light intensity elongates period and defers peak time of photosynthesis: a computational approach to circadian-clock-controlled photosynthesis in tomato
Source: Hortic Res. 2023 Apr 25;10(6):uhad077. doi: 10.1093/hr/uhad077 (PMC10261901; doi:10.1093/hr/uhad077)
Supplement: Web_Material_uhad077 [file web_material_uhad077.zip › Figure S2.pdf]

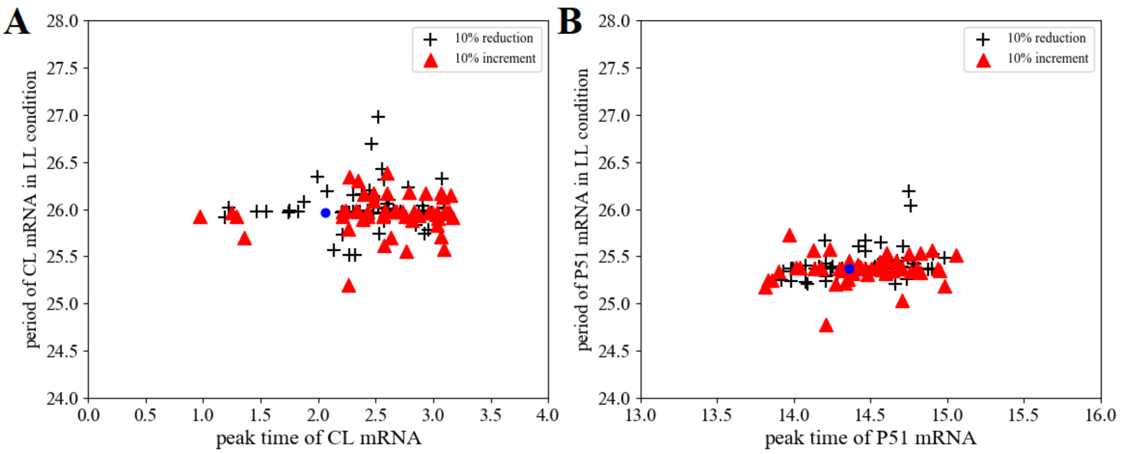

Figure S2. The change in the phase and period of CL and P51 mRNA in constant mediate-intensity light conditions measured under 10% increase and decrease of each parameter.

The robustness of the model to parameter variations was measured under 10 % changes of each parameter. The black pluses represent the parameter variations under 10% reduction. The red triangles denote the parameter variations under 10% increment. And the blue node indicates the original value.
