# Supplementary material for: Low light intensity elongates period and defers peak time of photosynthesis: a computational approach to circadian-clock-controlled photosynthesis in tomato
Source: Hortic Res. 2023 Apr 25;10(6):uhad077. doi: 10.1093/hr/uhad077 (PMC10261901; doi:10.1093/hr/uhad077)
Supplement: Web_Material_uhad077 [file web_material_uhad077.zip › Figure S3.pdf]

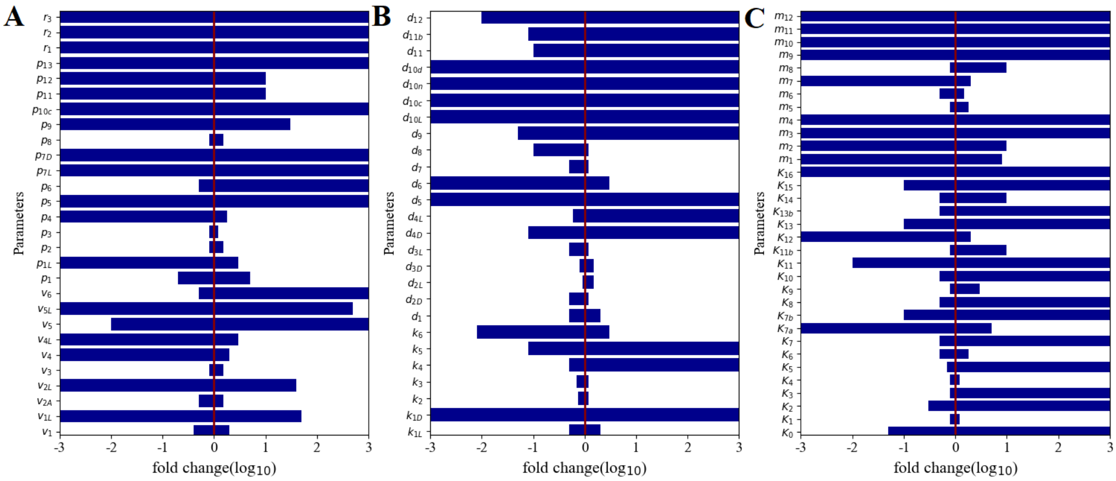

Figure S3. Sensitivity analysis for the phase and period of six genes under constant mediate light intensity.

In order to assess the sensitivity of the model, we determined for each parameter, one at a time, the specific range of expression peak time and the period occurrence exposure to free-running condition, with the base values listed in Table S2. In each sensitivity analysis, every parameter ranged from 10^{-3} to 10^{3} multiples of the basal values. 82 parameters are listed in the vertical axis. The horizontal axis shows the logarithmic scale of values from -3 to 3.
