# Supplementary material for: Low light intensity elongates period and defers peak time of photosynthesis: a computational approach to circadian-clock-controlled photosynthesis in tomato
Source: Hortic Res. 2023 Apr 25;10(6):uhad077. doi: 10.1093/hr/uhad077 (PMC10261901; doi:10.1093/hr/uhad077)
Supplement: Web_Material_uhad077 [file web_material_uhad077.zip › Figure S4.pdf]

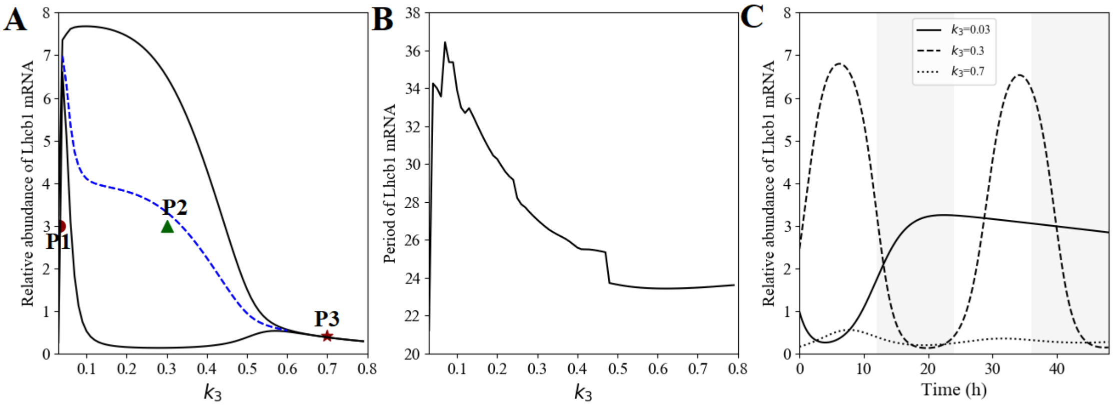

Figure S4. Bifurcation analysis of steady state and sustained oscillation in *Lhcb1* expression as well as the periods under oscillation areas.

The bifurcation diagram indicates *Lhcb1* mRNA oscillations as a function of the P51 mRNA degradation rate k_3. (A) *Lhcb1* mRNA level oscillates in gourd-shaped area and the blue dotted line represents the unstable equilibrium. (B) Periods of *Lhcb1* mRNA oscillations correspond to each bifurcation diagram shown in panel A. Time evolutions of *Lhcb1* mRNA were simulated by eq. (22) at point P1 ($k_3 = 0.03$, D), point P2 ($k_3 = 0.3$, D) and point P3 ($k_3 = 0.7$, D).
