# Supplementary material for: Low light intensity elongates period and defers peak time of photosynthesis: a computational approach to circadian-clock-controlled photosynthesis in tomato
Source: Hortic Res. 2023 Apr 25;10(6):uhad077. doi: 10.1093/hr/uhad077 (PMC10261901; doi:10.1093/hr/uhad077)
Supplement: Web_Material_uhad077 [file web_material_uhad077.zip › Figure S5.pdf]

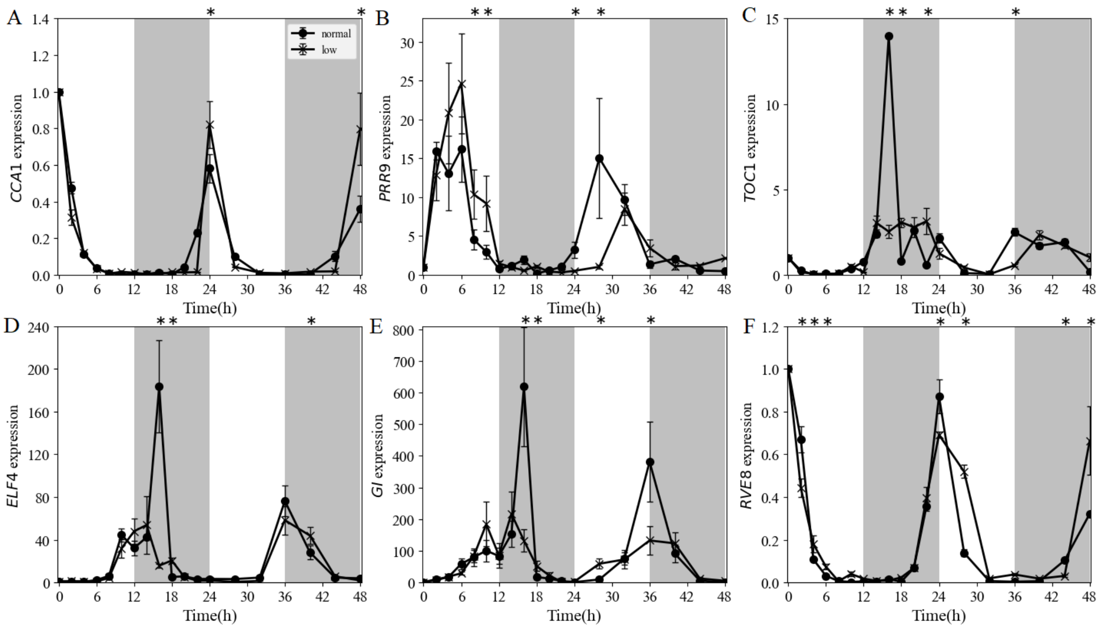

Figure S5. Experimental expression profiles of the main six circadian genes under 12L:12D cycles with low and high intensity.

Experimentally measured mRNA levels of (A) *CCA1*, (B) *PRR9*, (C) *TOC1*, (D) *ELF4*, (E) *GI* and (F) *RVE8*. The data was obtained by RT-qPCR (see the Material and Methods section). Symbols × and ● denote the tomato seedlings under low and normal intensity, respectively. Data are mean ± standard deviation for three biological replicates. Expression levels at diverse moments are statistically different marked by symbol ∗ ($P < 0.05$, by Wilcoxon rank testing).
