# Supplementary material for: Low light intensity elongates period and defers peak time of photosynthesis: a computational approach to circadian-clock-controlled photosynthesis in tomato
Source: Hortic Res. 2023 Apr 25;10(6):uhad077. doi: 10.1093/hr/uhad077 (PMC10261901; doi:10.1093/hr/uhad077)
Supplement: Web_Material_uhad077 [file web_material_uhad077.zip › Figure S6.pdf]

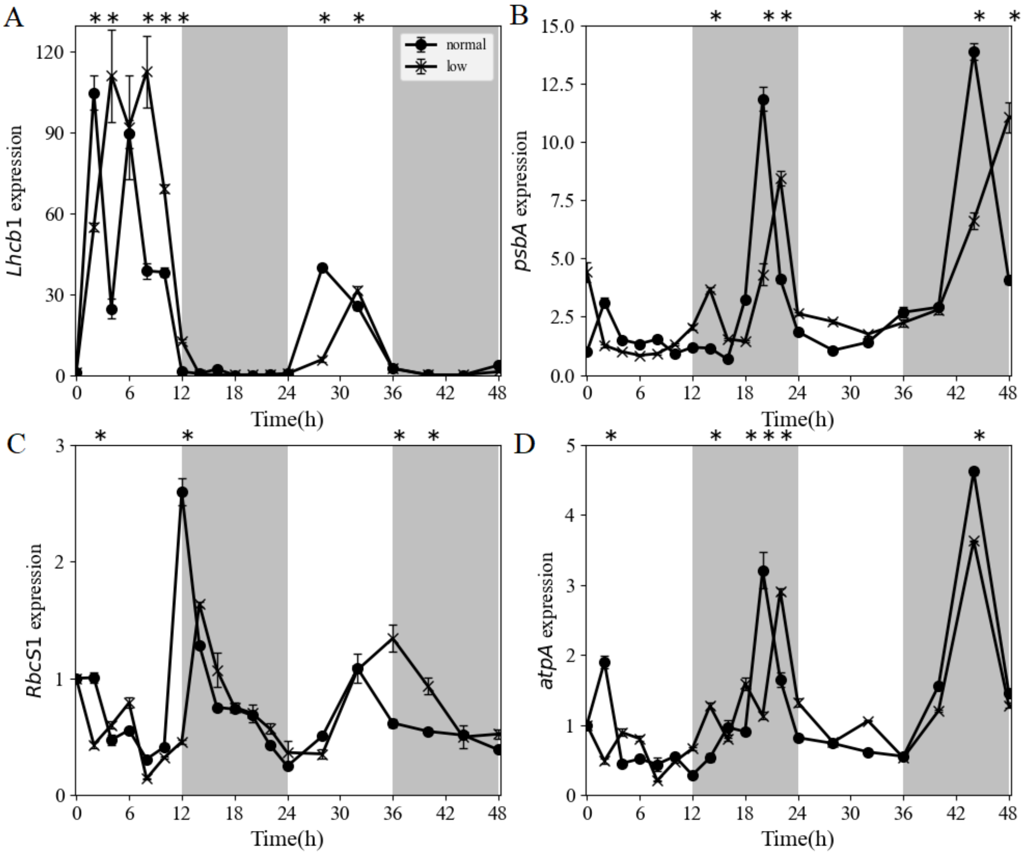

Fig.S6 Experimental expression profiles of the photosynthetic related genes under 12L:12D cycles with low and normal intensity.

The experimental data was obtained by RT-qPCR (see Material and Methods). Symbol × and ● denote the tomato seedlings under low and normal intensity, respectively. Data are mean ± standard deviation for three biological replicates. Expressions at diverse moments are statistically different marked by symbol ∗ ($P < 0.05$, by Wilcoxon rank testing).
