# Supplementary material for: Low light intensity elongates period and defers peak time of photosynthesis: a computational approach to circadian-clock-controlled photosynthesis in tomato
Source: Hortic Res. 2023 Apr 25;10(6):uhad077. doi: 10.1093/hr/uhad077 (PMC10261901; doi:10.1093/hr/uhad077)
Supplement: Web_Material_uhad077 [file web_material_uhad077.zip › Figure S7.pdf]

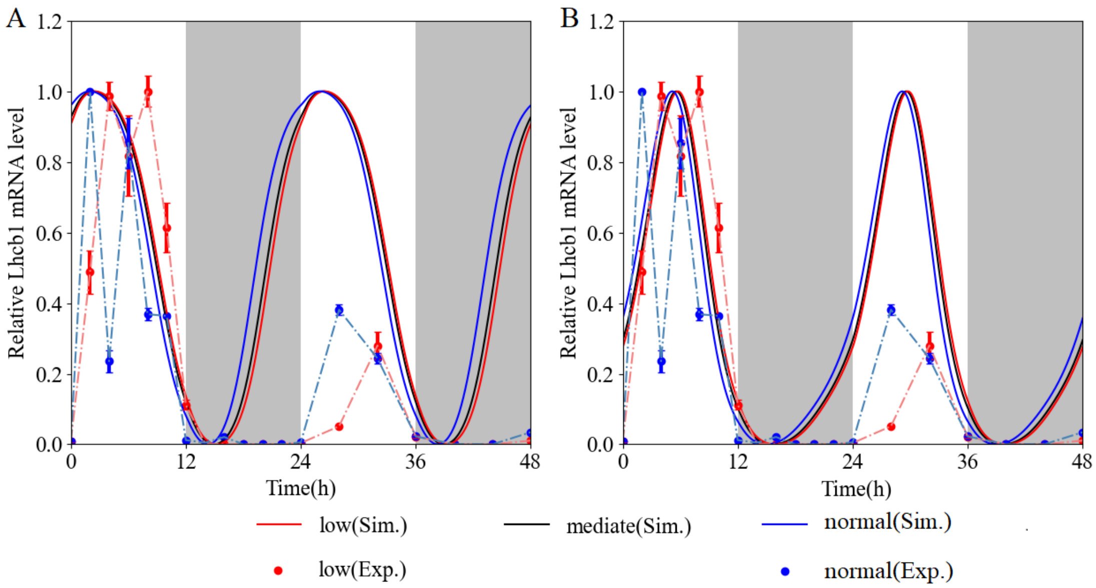

Fig. S7 Dynamic behaviors of *Lhcb1* controlled by circadian clocks in wild type compared with the control model.

(A) The simulated expression of *Lhcb1* modeled by CCA1 activation. (B) The simulated expression of *Lhcb1* under cooperative regulation of promotion by CCA1 and inhibition by GI. The red dots and blue dots are the corresponding expression profiles of *Lhcb1* under low light and normal light intensities, respectively. The number of biological replicates is 3. Values are normalized to their respective maximum. The grey bands denote the dark, while the white bands represent the light.
