# Supplementary material for: Low light intensity elongates period and defers peak time of photosynthesis: a computational approach to circadian-clock-controlled photosynthesis in tomato
Source: Hortic Res. 2023 Apr 25;10(6):uhad077. doi: 10.1093/hr/uhad077 (PMC10261901; doi:10.1093/hr/uhad077)
Supplement: Web_Material_uhad077 [file web_material_uhad077.zip › Figure S8.pdf]

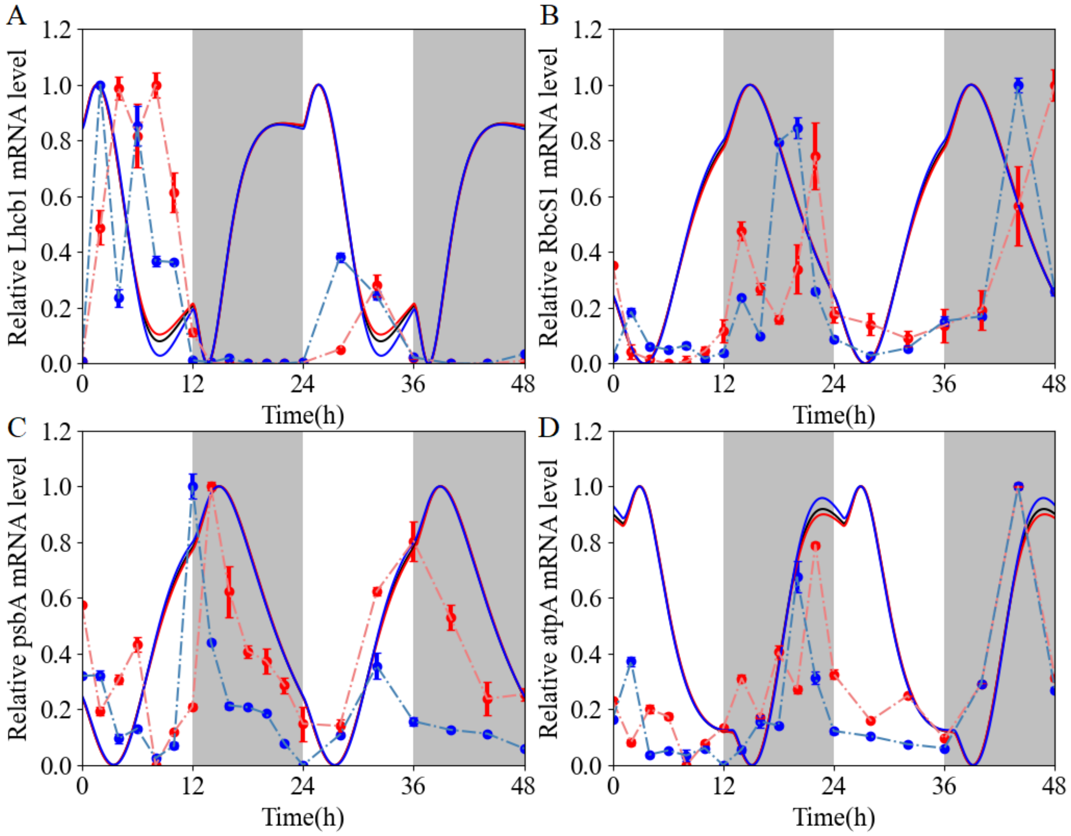


**Fig. S8 Dynamic behaviors of photosynthetic genes controlled by four pair genes in the compact model [25] in wild type.**

The simulated expression of *Lhcb1* (A), *RcbS1* (B), *psbA* (C), *atpA* (D). The red dots and blue dots are the corresponding expression profiles under low light and normal light intensities, respectively (Fig. 5).
