# Supplementary material for: Low light intensity elongates period and defers peak time of photosynthesis: a computational approach to circadian-clock-controlled photosynthesis in tomato
Source: Hortic Res. 2023 Apr 25;10(6):uhad077. doi: 10.1093/hr/uhad077 (PMC10261901; doi:10.1093/hr/uhad077)
Supplement: Web_Material_uhad077 [file web_material_uhad077.zip › Supplementary Material File.docx]

Low light intensity elongates period and defers peak time of photosynthesis: a computational approach for the circadian-clock-controlled photosynthesis in tomato

***Supplementary Material***

Ting Huang ^1,#^, Hui Liu ^1,#^, Jian-Ping Tao^1,#^, Jiaqi Zhang ^1^, Tongmin Zhao ^3^, Xilin Hou ^1^, Aisheng Xiong ^1,^* and Xiong You ^2,^*

Page

[1. Kinetic equations for characterizing photosynthesis through tomato circadian clock pathway under different light intensities 2](#_Toc115629789)

[2. Model parameterization and sensitivity analysis 3](#_Toc115629790)

[2.1 Model parameterization 3](#_Toc115629791)

[2.2 Simulating annealing algorithm 4](#_Toc115629792)

[2.3 Sensitivity analysis of parameters 4](#_Toc115629793)

[3. Numerical simulation 5](#_Toc115629794)

[4. Computer codes used for numerical simulation 5](#_Toc115629795)

[5. Reference 6](#_Toc115629796)

# 1. Kinetic equations for characterizing photosynthesis through tomato circadian clock pathway under different light intensities

Based on the compact circadian model by De Caluwé et al. [1] and subsequent modifications of the circadian network [2,3], we developed a light-intensity-entrainment model with six pairs of circadian genes and a photosynthesis-related gene *Lhcb1* as output variable. The gene regulation diagram was presented in Figure 1. The model consists of ordinary differential equations listed as eqs. (S1)—(S22). Each description of variables and parameters can be acquired in Section 3 below.

The time evolution of the mRNA and protein levels in circadian clock involving in the variables CL (CCA1/LHY), P97 (PRR9/PRR7), P51 (PRR5/TOC1), EL (ELF4/LUX), GI, RVE8, and the activity of P are governed by the following differential equations:

$\frac{\text{d}[\mathrm{MCL}]}{\text{d}t}=(v_{1}+v_{1L}L\left( t \right)\left[ P \right]\frac{I}{I_{A}*I^{2}+I_{B}*I+I_{C}})\frac{1}{1+\left( \frac{\left[ \mathrm{CL} \right]}{K_{0}} \right)^{2}+\left( \frac{\left[ P97 \right]}{K_{1}} \right)^{2}+\left( \frac{\left[ P51 \right]}{K_{2}} \right)^{2}}-(k_{1L}L\left( t \right)+k_{1D}D\left( t \right))[\mathrm{MCL}]$ (S1)

$\frac{\text{d}[\mathrm{CL}]}{\text{d}t}=(p_{1}+p_{1L}L\left( t \right))[\mathrm{MCL}]-d_{1}[\mathrm{CL}]$ (S2)

$\frac{d[MP97]}{dt}=(v_{2L}L\left( t \right)[P]\frac{I}{I_{A}*I^{2}+I_{B}*I+I_{C}}+v_{2A})\frac{1}{1+\left( \frac{\left[ P51 \right]}{K_{3}} \right)^{2}+\left( \frac{\left[ \mathrm{EL} \right]}{K_{4}} \right)^{2}+\left( \frac{\left[ \mathrm{CL} \right]}{K_{5}} \right)^{2}}-k_{2}[MP97]$ (S3)

$\frac{\text{d}[P97]}{\text{d}t}=p_{2}[MP97]-(d_{2D}D\left( t \right)+d_{2L}L\left( t \right))[P97]$  (S4)

$\frac{d[MP51]}{dt}=v_{3}\frac{\left( \frac{\left[ \mathrm{RL} \right]}{K_{7a}} \right)^{2}}{1+\left( \frac{\left[ \mathrm{RL} \right]}{K_{7a}} \right)^{2}}\frac{\left( \frac{\left[ \mathrm{GI} \right]}{K_{7c}} \right)^{2}}{1+\left( \frac{\left[ \mathrm{GI} \right]}{K_{7c}} \right)^{2}}\frac{1}{1+\left( \frac{\left[ \mathrm{CL} \right]}{K_{6}} \right)^{2}+\left( \frac{\left[ P51 \right]}{K_{7}} \right)^{2}+\left( \frac{\left[ \mathrm{EC} \right]}{K_{7b}} \right)^{2}}-k_{3}[MP51]$ (S5)

$\frac{\text{d}[P51]}{\text{d}t}=p_{3}\left[ MP51 \right]-(m_{1}+m_{2}D)[P51](\left[ \mathrm{ZTL} \right]+[\mathrm{ZG}])-(d_{3D}D\left( t \right)+d_{3L}L\left( t \right))[P51]$ (S6)

$\frac{d[\mathrm{MEL}]}{dt}=(v_{4}+v_{4L}L\left( t \right)[P]\frac{I}{I_{A}*I^{2}+I_{B}*I+I_{C}})\frac{1}{1+\left( \frac{\left[ \mathrm{CL} \right]}{K_{8}} \right)^{2}+\left( \frac{\left[ P51 \right]}{K_{9}} \right)^{2}+\left( \frac{\left[ \mathrm{EL} \right]}{K_{10}} \right)^{2}+\left( \frac{\left[ \mathrm{EC} \right]}{K_{11}} \right)^{2}}\frac{\left( \frac{\left[ R8 \right]}{K_{11b}} \right)^{2}}{1+\left( \frac{\left[ R8 \right]}{K_{11b}} \right)^{2}}-k_{4}[\mathrm{MEL}]$ (S7)

$\frac{\text{d}[\mathrm{EL}]}{\text{d}t}=p_{4}[\mathrm{MEL}]-(d_{4D}D\left( t \right)+d_{4L}L\left( t \right))[\mathrm{EL}]$ (S8)

$\frac{\text{d}[\mathrm{MGI}]}{\text{d}t}=\left( v_{5L}L\left( t \right)\left[ P \right]\frac{I}{I_{A}*I^{2}+I_{B}*I+I_{C}}+v_{5} \right)\frac{1}{1+\left( \frac{\left[ \mathrm{CL} \right]}{K_{12}} \right)^{2}+\left( \frac{\left[ \mathrm{EC} \right]}{K_{13}} \right)^{2}+\left( \frac{\left[ P97 \right]}{K_{13b}} \right)^{2}+\left( \frac{\left[ P51 \right]}{K_{13c}} \right)^{2}}-k_{5}[\mathrm{MGI}]$ (S9)

$\frac{\text{d}[\mathrm{GI}]}{\text{d}t}=p_{5}\left[ \mathrm{MGI} \right]-m_{3}L\left( t \right)\left[ \mathrm{ZTL} \right]\left[ \mathrm{GI} \right]+m_{4}D\left( t \right)\left[ \mathrm{ZG} \right]-d_{5}[\mathrm{GI}]$ (S10)

$\frac{\text{d}[MR8]}{\text{d}t}=v_{6}\frac{1}{1+\left( \frac{\left[ P97 \right]}{K_{14}} \right)^{2}+\left( \frac{\left[ P51 \right]}{K_{15}} \right)^{2}}-k_{6}[MR8]$ (S11)

$\frac{\text{d}[R8]}{\text{d}t}=p_{6}\left[ MR8 \right]-m_{5}\left[ R8 \right]\left[ LNK1 \right]+m_{6}\left[ \mathrm{RL} \right]-d_{6}[R8]$ (S12)

$\frac{\text{d}[LNK1]}{\text{d}t}=(p_{7L}L(t)+p_{7D}D\left( t \right))\frac{\left( \frac{\left[ \mathrm{EC} \right]}{K_{16}} \right)^{2}}{1+\left( \frac{\left[ \mathrm{EC} \right]}{K_{16}} \right)^{2}}-m_{7}\left[ R8 \right]\left[ LNK1 \right]+m_{8}\left[ \mathrm{RL} \right]-d_{7}[LNK1]$ (S13)

$\frac{\text{d}[\mathrm{RL}]}{\text{d}t}=p_{8}\left[ R8 \right]\left[ LNK1 \right]-d_{8}\left[ \mathrm{RL} \right]$ (S14)

$\frac{\text{d}[\mathrm{EC}]}{\text{d}t}=p_{9}\left[ \mathrm{EL} \right]-m_{9}\left[ \mathrm{EC} \right]\left[ COP1n \right]-m_{10}\left[ \mathrm{EC} \right]\left[ COP1d \right]-d_{9}[\mathrm{EC}]$ (S15)

$\frac{\text{d}[COP1c]}{\text{d}t}=p_{10c}-r_{1}\left[ COP1c \right]-(d_{10L}L\left( t \right)+d_{10c})[COP1c]$ (S16)

$\frac{\text{d}[COP1n]}{\text{d}t}=r_{1}\left[ COP1c \right]-\left( r_{2}L\left( t \right)\left[ P \right]+r_{3} \right)\left[ COP1n \right]-d_{10n}[COP1n]$ (S17)

$\frac{\text{d}[COP1d]}{\text{d}t}=\left( r_{2}L\left( t \right)\left[ P \right]+r_{3} \right)\left[ COP1n \right]-d_{10d}[COP1d]$ (S18)

$\frac{\text{d}[\mathrm{ZTL}]}{\text{d}t}=p_{11}-m_{11}L\left( t \right)\left[ \mathrm{ZTL} \right]\left[ \mathrm{GI} \right]+m_{12}D\left( t \right)\left[ \mathrm{ZG} \right]-d_{11}[\mathrm{ZTL}]$ (S19)

$\frac{\text{d}[\mathrm{ZG}]}{\text{d}t}=m_{11}L\left( t \right)\left[ \mathrm{ZTL} \right]\left[ \mathrm{GI} \right]-m_{12}D\left( t \right)\left[ \mathrm{ZG} \right]-d_{11b}[\mathrm{ZG}]$ (S20)

$\frac{\text{d}[P]}{\text{d}t}=p_{12}\left( 1-[P] \right)D\left( t \right)-d_{12}[P]L\left( t \right)$ (S21)

The functions $L$, $D$ represent light and darkness, respectively. $L = 1$ and $D = 0$ when the system is in light, whereas$L = 0$ and $D = 1$ when the system is in dark. If $I_{A}=0$, $I_{B}=1$, $I_{C}=0$, the system was under intermediate light intensity; $\frac{I}{I_{A}*I^{2}+I_{B}*I+I_{C}}>1$ ($\frac{I}{I_{A}*I^{2}+I_{B}*I+I_{C}}<1$) indicate the system is under high (low) light intensity.

The time evolution of the photosynthesis-response gene *Lhcb1*, *psbA*, *RbcS1*, *atpA* mRNA has two alternative equations:

$\frac{\text{d}[MLhcb1]}{\text{d}t}=v_{7}\frac{\left( \frac{\left[ \mathrm{CL} \right]}{K_{17}} \right)^{2}}{1+\left( \frac{\left[ \mathrm{CL} \right]}{K_{17}} \right)^{2}}\cdot\frac{1}{1+\left( \frac{\left[ \mathrm{GI} \right]}{K_{18}} \right)^{2}}-k_{7}[MLhcb1]$  (S22)

$\frac{\text{d}[MLhcb1]}{\text{d}t}=v_{7}\frac{\left( \frac{\left[ \mathrm{CL} \right]}{K_{17}} \right)^{2}}{1+\left( \frac{\left[ \mathrm{CL} \right]}{K_{17}} \right)^{2}}-k_{7}[MLhcb1]$ (S22b)

$\frac{\text{d}[Lhcb1]}{\text{d}t}=p_{13}[MLhcb1]-d_{13}[Lhcb1]$ (S23)

$\frac{\text{d}[\mathrm{MpsbA}]}{\text{d}t}=v_{8}\frac{1}{1+\left( \frac{\left[ \mathrm{CL} \right]}{K_{19}} \right)^{2}}-k_{8}[\mathrm{MpsbA}]$ (S24)

$\frac{\text{d}[\mathrm{psbA}]}{\text{d}t}=p_{14}[\mathrm{MpsbA}]-d_{14}[\mathrm{psbA}]$ (S25)

$\frac{\text{d}[MRbcS1]}{\text{d}t}=v_{9}\frac{1}{1+\left( \frac{\left[ \mathrm{CL} \right]}{K_{20}} \right)^{2}}-k_{9}[MRbcS1]$ (S26)

$\frac{\text{d}[RbcS1]}{\text{d}t}=p_{15}[MRbcS1]-d_{15}[RbcS1]$ (S27)

$\frac{\text{d}[\mathrm{MatpA}]}{\text{d}t}=v_{10}\frac{1}{1+\left( \frac{\left[ \mathrm{CL} \right]}{K_{21}} \right)^{2}}-k_{10}[\mathrm{MatpA}]$ (S28)

$\frac{\text{d}[\mathrm{atpA}]}{\text{d}t}=p_{16}[\mathrm{MatpA}]-d_{16}[\mathrm{atpA}]$ (S29)

# 2. Model parameterization and sensitivity analysis

## 2.1 Model parameterization

Model development is followed by model calibration, in which experimental data are often capitalized to estimate the kinetic parameters of the model. In the one hand, some of these parameters can be determined by a published research work. On the other hand, some database that stores comprehensive information on biochemical reactions and their kinetic properties are extremely valuable for parameter determination. However, there are few existing dynamics databases related to the light response of plant circadian rhythm, so the most values of dynamics parameters are uncertain.

Kinetic data generally contain the characteristic of steady-state, amplitude, phase etc. They are the end-point, peak, peak-time of perturbation experiments or time course data. Since time course data describes a more detailed dynamic process, it is preferable for the parameter estimation process and time series analysis. This enables us to construct a cost function for good fitness to the kinetic characters. The cost function is defined by

$$\delta=\sum_{i=G} \left( \frac{y_{i}-\bar{y_{i}}}{\bar{y_{i}}} \right)^{2}+\sum_{i=G} \left( \frac{\phi_{i}-\phi_{i_{0}}}{\phi_{i_{0}}} \right)^{2}+\sum_{i=C,T} \left( \frac{{PI}_{i}-{PI}_{i_{0}}}{{PI}_{i_{0}}} \right)^{2}$$

$:=\delta_{SSE}+\delta_{\phi}+\delta_{PI}$.

The cost function is a sum of terms. We now discuss these terms in turn: $G$ genes denote the genes *CCA1*, *PRR9*, *TOC1*, *ELF4*, *GI* and *RVE8*; $\delta_{SSE}$ measures the difference between the model simulated expression and the experimental expression extracted from the published data [4]; The following term $\delta_{\phi}$ scales the peak times (phase) between simulated and experimentally observed data; the last term estimates the difference between the experimental period and the mean period of the oscillation in mRNA levels of $G$ genes exhibited by the model.

## 2.2 Simulating annealing algorithm

The first step: data (expressions, periods, and phases) were redrawn and calculated and parameter values were generated by sobol sequences with $92\times{10}^{6}$ dimension.

The second step: we started the process with a set of parameters taking the first column of 92 parameters from the generated parameter set, and selected them by column in turn.

The third step: for $1\leq i\leq{10}^{6}$ do, at each step 𝑖, we perturbed a randomly chosen parameter by adding to it a Gaussian random number with mean 0 and variance 1, always checking that no parameter became negative.

The fourth step: for the perturbed set of parameters, we computed the cost function $\delta_{i}$, compared it with the energy of the old parameter set $\delta_{i-1}$, and accepted the set with probability

$${Prob}_{acc}=\left\{ \begin{matrix} 1 \\ \frac{\delta_{i-1}}{\delta_{i}}e^{-(i+1)} \end{matrix} \right.\begin{matrix} ,if\frac{\delta_{i}}{\delta_{i-1}}<1 \\ ,f\frac{\delta_{i}}{\delta_{i-1}}\geq1 \end{matrix}$$

The model made dynamic predictions under HI and LI conditions in the accepted parameter sets and finally found an optimal parameter set by comparing with the experimental data.

## 2.3 Sensitivity analysis of parameters

Local sensitivity analysis (LSA) and global sensitivity analysis (GSA) are two major approaches to analyzing sensitivity [5]. And various techniques are widely applied including differential sensitivity analysis, one at a time sensitivity measures, factorial analysis, correlation analysis, regression analysis and subjective sensitivity analysis in different sensitivity methods. The most fundamental method with differentiation is one at a time sensitivity measure, in which varying parameter values are taken one at a time [6]. One at a time sensitivity measure is also known as a local analysis method as it aims to gain the addressed point estimation and not the entire distribution. Sensitivity analysis is often employed to change the parameters of model and observe the behaviors of the system. The steps used to conduct sensitivity analysis are listed below.

First step: The base case parameters are defined and a particular parameter value is tested for the sensitivity to be measured. All the other parameters of the model are kept constant.

Second step: The behavior of the variable output at different values of the changed parameter.

Third step: Find the percentage change in the output of parameters and the percentage change in the basal value.

Fourth step: The sensitivity of the parameter is calculated by dividing the percentage change in output by the percentage change in the basal value, where percentages greater than 100 are usually written in integer multiples.

This process of testing sensitivity for one input parameter (e.g., transcription, translation, and degradation rate) while keeping the rest of parameters constant is repeated until the sensitivity figure for each of the input parameters is obtained.

# 3. Numerical simulation

In general, nonlinear differential equations do not have analytical solution. Different numerical methods can help us to obtain numerical solutions with high accuracy and small errors. The most successful methods during more than half a century were the 4th order methods of Kutta [8]. Therefore, we choose a 4th order method (Gauss 2s4) in the model analysis. The numerical scheme and the corresponding Butcher table are given below.

Numerical scheme:

$$\left\{ \begin{matrix} Y_{1}=y_{n}+h\sum_{j=1}^{2} a_{1j}f(x_{n}+c_{j}h,Y_{j}) \\ Y_{2}=y_{n}+h\sum_{j=1}^{2} a_{2j}f(x_{n}+c_{j}h,Y_{j}) \\ y_{n+1}=y_{n}+h\sum_{i=1}^{2} b_{i}f(x_{n}+c_{j}h,Y_{i}) \end{matrix} \right.$$

Butcher table:

| $\frac{\boldsymbol{1}}{\boldsymbol{2}}\boldsymbol{-}\frac{\sqrt{\boldsymbol{3}}}{\boldsymbol{6}}$ | $\frac{\boldsymbol{1}}{\boldsymbol{4}}$ | $\frac{\boldsymbol{1}}{\boldsymbol{4}}\boldsymbol{-}\frac{\sqrt{\boldsymbol{3}}}{\boldsymbol{6}}$ |
| --- | --- | --- |
| $\frac{\boldsymbol{1}}{\boldsymbol{2}}\boldsymbol{+}\frac{\sqrt{\boldsymbol{3}}}{\boldsymbol{6}}$ | $\frac{1}{4}+\frac{\sqrt{3}}{6}$ | $\frac{1}{4}$ |
|  | $\frac{1}{2}$ | $\frac{1}{2}$ |

# 4. Computer codes used for numerical simulation

The codes for the numerical simulation (Fig. 2-8, Fig. S2-S7) of the model were attached as python format files.

# 5. Reference

1. De Caluwé J, Xiao Q, Hermans C, *et al*. A compact model for the complex plant circadian clock. *Front Plant Sci*. 2016; **7**:74.

2. Oakenfull RJ, Davis SJ. Shining a light on the Arabidopsis circadian clock. *Plant Cell Environ.* 2017; **40**:2571–85.

3. Creux N, Harmer S. Circadian Rhythms in Plants. *Cold Spring Harb Perspect Biol.* 2019; **11**: a034611.

4. Müller NA, Wijnen CL, Srinivasan A, *et al*. Domestication selected for deceleration of the circadian clock in cultivated tomato. *Nat Genet.* 2016; **48**: 89–93.

5. Hoops S, Hontecillas R, Abedi V, *et al*. Ordinary Differential Equations (ODEs) Based Modeling. Computational Immunology, Elsevier; 2016, p. 63–78.

6. Leloup JC, Goldbeter A. Modeling the mammalian circadian clock: sensitivity analysis and multiplicity of oscillatory mechanisms. *J Theor Biol.* 2004; **230**: 541–62.

7. Zhang RQ, Gonze D, Hou XL, *et al*. A computational model for﻿﻿ the cold response pathway in plants. *Frontiers Physiol.* 2020; **11**: 591073.

8. Hairer E, Lubich C, Wanner G. Geometric numerical integration: structure-preserving algorithms for ordinary differential equations. 2nd ed. Berlin ; New York: Springer; 2006.
