# Supplementary material for: Low light intensity elongates period and defers peak time of photosynthesis: a computational approach to circadian-clock-controlled photosynthesis in tomato
Source: Hortic Res. 2023 Apr 25;10(6):uhad077. doi: 10.1093/hr/uhad077 (PMC10261901; doi:10.1093/hr/uhad077)
Supplement: Web_Material_uhad077 [file web_material_uhad077.zip › Table S1.docx]

## Table S1. Core variables for tomato circadian clock effect on photosynthesis.

The first column lists the sequence of variables and the definition of each variable is given in the second column.

| Symbol | Definition |
| --- | --- |
| $\boldsymbol{[}\mathbf{MCL}\boldsymbol{]}$ | concentration of CCA1/LHY mRNA |
| $\boldsymbol{[}\mathbf{CL}\boldsymbol{]}$ | concentration of CCA1/LHY protein |
| $\boldsymbol{[}\mathbf{MP97}\boldsymbol{]}$ | concentration of PRR9/PRR7 mRNA |
| $\boldsymbol{[}\mathbf{P97}\boldsymbol{]}$ | concentration of PRR9/PRR7 protein |
| $\boldsymbol{[}\mathbf{MP51}\boldsymbol{]}$ | concentration of PRR5/TOC1 mRNA |
| $\boldsymbol{[}\mathbf{P51}\boldsymbol{]}$ | concentration of PRR5/TOC1 protein |
| $\boldsymbol{[}\mathbf{MEL}\boldsymbol{]}$ | concentration of ELF4/LUX mRNA |
| $\boldsymbol{[}\mathbf{EL}\boldsymbol{]}$ | concentration of ELF4/LUX protein |
| $\boldsymbol{[}\mathbf{MGI}\boldsymbol{]}$ | concentration of GI mRNA |
| $\boldsymbol{[}\mathbf{GI}\boldsymbol{]}$ | concentration of GI protein |
| $\boldsymbol{[}\mathbf{MR8}\boldsymbol{]}$ | concentration of RVE8 mRNA |
| $\boldsymbol{[}\mathbf{R8}\boldsymbol{]}$ | concentration of RVE8 protein |
| $\boldsymbol{[}\mathbf{LNK1}\boldsymbol{]}$ | concentration of LNK1 protein |
| $\boldsymbol{[}\mathbf{RL}\boldsymbol{]}$ | concentration of RVE8-LNK1 complex protein |
| $\boldsymbol{[}\mathbf{EC}\boldsymbol{]}$ | concentration of the evening complex |
| $\boldsymbol{[}\mathbf{COP1c}\boldsymbol{]}$ | concentration of cytoplasmic COP1 protein |
| $\boldsymbol{[}\mathbf{COP1n}\boldsymbol{]}$ | concentration of nuclear COP1 protein in the night |
| $\boldsymbol{[}\mathbf{COP1d}\boldsymbol{]}$ | concentration of nuclear COP1 protein in the day |
| $\boldsymbol{[}\mathbf{ZTL}\boldsymbol{]}$ | concentration of ZTL protein |
| $\boldsymbol{[}\mathbf{ZG}\boldsymbol{]}$ | the concentration of ZTL-GI complex protein |
| $\boldsymbol{P}$ | The activation of light-sensitive proteins |
| $\boldsymbol{[}\mathbf{MLhcb1}\boldsymbol{]}$ | the concentration of Lhcb1 mRNA |
| $\boldsymbol{[}\mathbf{Lhcb1}\boldsymbol{]}$ | the concentration of Lhcb1 protein |
| $\boldsymbol{[}\mathbf{M}\mathrm{psbA}\boldsymbol{]}$ | the concentration of $\mathbf{psbA}$ mRNA |
| $\boldsymbol{[}\mathrm{psbA}\boldsymbol{]}$ | the concentration of $\mathbf{psbA}$ protein |
| $\boldsymbol{[}\mathbf{M}RbcS1\boldsymbol{]}$ | the concentration of $\mathbf{RbcS1}$ mRNA |
| $\boldsymbol{[}RbcS1\boldsymbol{]}$ | the concentration of $\mathbf{RbcS1}$ protein |
| $\boldsymbol{[}\mathbf{M}\mathrm{atpA}\boldsymbol{]}$ | the concentration of $\mathbf{atpA}$ mRNA |
| $\boldsymbol{[}\mathrm{atpA}\boldsymbol{]}$ | the concentration of $\mathbf{atpA}$ protein |
