# Supplementary material for: Low light intensity elongates period and defers peak time of photosynthesis: a computational approach to circadian-clock-controlled photosynthesis in tomato
Source: Hortic Res. 2023 Apr 25;10(6):uhad077. doi: 10.1093/hr/uhad077 (PMC10261901; doi:10.1093/hr/uhad077)
Supplement: Web_Material_uhad077 [file web_material_uhad077.zip › Table S2.docx]

## Table S2. Basic parameter values used for numerical simulations.

The first column lists the sequence of basal parameters applied in eq. (S1) – (S29). The explanation of each parameter is given in the second column, followed by the corresponding values in the third column. The last column indicates the units of parameters taking part in the different equations.

| Parameter | Description | Value | Unit |
| --- | --- | --- | --- |
| $v_{1}$ | synthesis of *CCA1/LHY* mRNA | 4.6 | nM h^-1^ |
| $v_{1L}$ | light-induced synthesis of *CCA1/LHY* mRNA | 2.0 | nM h^-1^ |
| $v_{2A}$ | synthesis of *PRR9/PRR7* mRNA | 2.27 | nM h^-1^ |
| $v_{2L}$ | light-induced synthesis of *PRR9/PRR7* mRNA | 5.0 | nM h^-1^ |
| $v_{3}$ | synthesis of *PRR5/TOC1* mRNA | 1.5 | nM h^-1^ |
| $v_{4}$ | synthesis of *ELF4/LUX* mRNA | 2.47 | nM h^-1^ |
| $v_{4L}$ | light-induced synthesis of *ELF4/LUX* mRNA | 1.2 | nM h^-1^ |
| $v_{5}$ | synthesis of *GI* mRNA | 12.5 | nM h^-1^ |
| $v_{5L}$ | light-induced synthesis of *GI* mRNA | 7.56 | nM h^-1^ |
| $v_{6}$ | synthesis of *RVE8* mRNA | 3.3 | nM h^-1^ |
| $v_{7}$ | synthesis of *Lhcb1* mRNA | 12.6 | nM h^-1^ |
| $v_{8}$ | synthesis of *psbA* mRNA | 5.8 | nM h^-1^ |
| $v_{9}$ | synthesis of *RbcS1* mRNA | 6.7 | nM h^-1^ |
| $v_{10}$ | synthesis of *atpA* mRNA | 9.6 | nM h^-1^ |
| $p_{1}$ | synthesis of CCA1/LHY protein | 0.76 | h^-1^ |
| $p_{1L}$ | light-induced synthesis of CCA1/LHY protein | 0.42 | h^-1^ |
| $p_{2}$ | synthesis of PRR9/PRR7 protein | 1.01 | h^-1^ |
| $p_{3}$ | synthesis of PRR5/TOC1 protein | 0.64 | h^-1^ |
| $p_{4}$ | synthesis of ELF4/LUX protein | 1.01 | h^-1^ |
| $p_{5}$ | synthesis of GI protein | 0.51 | h^-1^ |
| $p_{6}$ | synthesis of RVE8 protein | 4 | h^-1^ |
| $p_{7L}$ | light-induced synthesis of LNK1 protein | 1.3 | h^-1^ |
| $p_{7D}$ | dark-induced synthesis of LNK1 protein | 3.3 | h^-1^ |
| $p_{8}$ | synthesis of RVE8-LNK1 complex protein | 0.2 | h^-1^ |
| $p_{9}$ | synthesis of evening complex protein | 0.3 | h^-1^ |
| $p_{10c}$ | synthesis of cytoplasmic COP1 protein | 0.23 | h^-1^ |
| $p_{11}$ | synthesis of ZTL protein | 0.14 | h^-1^ |
| $p_{12}$ | synthesis of light-sensitive protein P | 0.13 | h^-1^ |
| $r_{1}$ | COP1 protein [transportation](javascript:;) from the cytoplasm to the nucleus | 0.6 | h^-1^ |
| $r_{2}$ | [nuclear](javascript:;) COP1 protein transformation from night form to day | 2 | h^-1^ |
| $r_{3}$ | [nuclear](javascript:;) COP1 protein transformation from day form to night | 0.1 | h^-1^ |
| $k_{1L}$ | *CCA1*/*LHY* mRNA degradation (light) | 0.53 | h^-1^ |
| $k_{1D}$ | *CCA1*/*LHY* mRNA degradation (dark) | 0.21 | h^-1^ |
| $k_{2}$ | *PRR9/PRR7* mRNA degradation | 0.35 | h^-1^ |
| $k_{3}$ | *PRR5/TOC1* mRNA degradation | 0.72 | h^-1^ |
| $k_{4}$ | *ELF4/LUX* mRNA degradation | 0.04 | h^-1^ |
| $k_{5}$ | *GI* mRNA degradation | 3.4 | h^-1^ |
| $k_{6}$ | *RVE8* mRNA degradation | 1.5 | h^-1^ |
| $k_{7}$ | *Lhcb1* mRNA degradation | 1.6 | h^-1^ |
| $k_{8}$ | *psbA* mRNA degradation | 0.52 | h^-1^ |
| $k_{9}$ | *RbcS1* mRNA degradation | 0.15 | h^-1^ |
| $k_{10}$ | *atpA* mRNA degradation | 0.63 | h^-1^ |
| $d_{1}$ | CCA1/LHY protein degradation | 0.48 | h^-1^ |
| $d_{2D}$ | PRR9/PRR7 protein degradation (dark) | 0.5 | h^-1^ |
| $d_{2L}$ | PRR9/PRR7 protein degradation (light) | 0.29 | h^-1^ |
| $d_{3D}$ | PRR5/TOC1 protein degradation (dark) | 0.48 | h^-1^ |
| $d_{3L}$ | PRR5/TOC1 protein degradation (light) | 0.78 | h^-1^ |
| $d_{4D}$ | ELF4/LUX protein degradation (dark) | 1.21 | h^-1^ |
| $d_{4L}$ | ELF4/LUX protein degradation (light) | 0.38 | h^-1^ |
| $d_{5}$ | GI protein degradation | 0.2 | h^-1^ |
| $d_{6}$ | RVE8 protein degradation | 1.5 | h^-1^ |
| $d_{7}$ | LNK1 protein degradation | 0.4 | h^-1^ |
| $d_{8}$ | RVE8- LNK1 complex degradation | 0.3 | h^-1^ |
| $d_{9}$ | Evening complex degradation | 5.2 | h^-1^ |
| $d_{10L}$ | cytoplasmic COP1 protein degradation (light) | 0.3 | h^-1^ |
| $d_{10c}$ | cytoplasmic COP1 protein degradation | 3 | h^-1^ |
| $d_{10n}$ | [nuclear](javascript:;) COP1 protein (night form) degradation | 0.1 | h^-1^ |
| $d_{10d}$ | [nuclear](javascript:;) COP1 protein (day form) degradation | 0.3 | h^-1^ |
| $d_{11}$ | ZTL protein degradation | 6.6 | h^-1^ |
| $d_{11b}$ | ZTL-GI complex degradation | 0.8 | h^-1^ |
| $d_{12}$ | $P$ protein degradation | 0.5 | h^-1^ |
| $K_{0}$ | inhibition of *CCA1/LHY* by CCA1/LHY | 5.07 | nM |
| $K_{1}$ | inhibition of *CCA1/LHY* by PRR9/PRR7 | 0.3 | nM |
| $K_{2}$ | inhibition of *CCA1/LHY* by PRR5/TOC1 | 1.3 | nM |
| $K_{3}$ | inhibition of *PRR9/PRR7* by PRR5/TOC1 | 0.4 | nM |
| $K_{4}$ | inhibition of *PRR9/PRR7* by ELF4/LUX | 0.62 | nM |
| $K_{5}$ | inhibition of *PRR9/PRR7* by CCA1/LHY | 1.2 | nM |
| $K_{6}$ | inhibition of *PRR5/TOC1* by CCA1/LHY | 0.46 | nM |
| $K_{7}$ | inhibition of *PRR5/TOC1* by PRR5/TOC1 | 5 | nM |
| $K_{7a}$ | activation of *PRR5/TOC1* by RVE8-LNK1 complex | 1 | nM |
| $K_{7b}$ | inhibition of *PRR5/TOC1* by EC | 3.5 | nM |
| $K_{7b}$ | activation of *PRR5/TOC1* by GI | 0.5 | nM |
| $K_{8}$ | inhibition of *ELF4/LUX* by CCA1/LHY | 1.36 | nM |
| $K_{9}$ | inhibition of *ELF4/LUX* by PRR5/TOC1 | 0.9 | nM |
| $K_{10}$ | inhibition of *ELF4/LUX* by ELF4/LUX | 1.9 | nM |
| $K_{11}$ | inhibition of *ELF4/LUX* by EC | 5 | nM |
| $K_{11b}$ | activation of *ELF4/LUX* by RVE8 | 4.5 | nM |
| $K_{12}$ | inhibition of *GI* by CCA1/LHY | 0.4 | nM |
| $K_{13}$ | inhibition of *GI* by EC | 3 | nM |
| $K_{13b}$ | inhibition of *GI* by PRR9/PRR7 | 2.5 | nM |
| $K_{13c}$ | inhibition of *GI* by PRR5/TOC1 | 1.5 | nM |
| $K_{14}$ | inhibition of *RVE8* by PRR9/PRR7 | 2.1 | nM |
| $K_{15}$ | inhibition of *RVE8* by PRR5/TOC1 | 5 | nM |
| $K_{16}$ | activation of LNK1 by EC | 1 | nM |
| $K_{17}$ | activation of *Lhcb1* by CCA1/LHY | 0.5 | nM |
| $K_{18}$ | inhibition of *Lhcb1* by GI | 0.8 | nM |
| $K_{19}$ | inhibition of *psbA* by CCA1 | 0.3 | nM |
| $K_{20}$ | inhibition of *RbcS1* by CCA1 | 0.5 | nM |
| $K_{21}$ | inhibition of *atpA* by CCA1 | 1.12 | nM |
| $m_{1}$ | PRR5/TOC1 degradation induced by ZTL and ZG | 0.3 | h^-1^ |
| $m_{2}$ | dark-induced PRR5/TOC1 degradation by ZTL and ZG | 0.7 | h^-1^ |
| $m_{3}$ | light-induced GI degradation by ZTL | 3.4 | h^-1^ |
| $m_{4}$ | dark-induced GI synthesis by ZG | 0.1 | h^-1^ |
| $m_{5}$ | RVE8 degradation by LNK1 | 0.5 | h^-1^ |
| $m_{6}$ | RVE8 synthesis by RL | 0.6 | h^-1^ |
| $m_{7}$ | LNK1 degradation by RVE8 | 0.1 | h^-1^ |
| $m_{8}$ | LNK1 synthesis by RL | 1.3 | h^-1^ |
| $m_{9}$ | EC degradation by COP1n | 0.1 | h^-1^ |
| $m_{10}$ | EC degradation by COP1d | 0.8 | h^-1^ |
| $m_{11}$ | ZG synthesis by ZTL and GI | 3.4 | h^-1^ |
| $m_{12}$ | dark-induced ZG degradation | 0.1 | h^-1^ |
