# Supplementary material for: Low light intensity elongates period and defers peak time of photosynthesis: a computational approach to circadian-clock-controlled photosynthesis in tomato
Source: Hortic Res. 2023 Apr 25;10(6):uhad077. doi: 10.1093/hr/uhad077 (PMC10261901; doi:10.1093/hr/uhad077)
Supplement: Web_Material_uhad077 [file web_material_uhad077.zip › Table S3.docx]

| **Table S3 Primers of core circadian elements and photosynthetic genes used in this paper.** | | |
| --- | --- | --- |
| Primer | Sequence (from 5' to 3') | Gene ID |
| SlCCA1-qRT-F | ATGCAAGTGGATGCCTGTCA | Solyc10g005080.2 |
| SlCCA1-qRT-R | GAGCGATCTACCTGCTTCCC |  |
| SlPRR9-qRT-F | CCGGTTGACAGGGTAAGGTC | Solyc10g005030.2 |
| SlPRR9-qRT-R | GACTGTGATCGAGGGCTCTG |  |
| SlTOC1-qRT-F | CTGCACAATAAAGCCCAGCG | Solyc03g115770.2 |
| SlTOC1-qRT-R | ATTTCGCCAGGTCGTAAGCA |  |
| SlELF3-qRT-F | AGATTCTGAGTTGCACGCCA | Solyc11g070100.1 |
| SlELF3-qRT-R | AAAATGAGGCTGAGGCCGAA |  |
| SlELF4-qRT-F | TCGTGACAACTCTACCGTGG | Solyc06g051680.1 |
| SlELF4-qRT-R | CGTTCTGCACCATGCTATCG |  |
| SlLUX-qRT-F | GTGATGGTTACGCCGGAGAT | Solyc06g005680.2 |
| SlLUX-qRT-R | CTCCGGCGGGATTAAAACCT |  |
| SlGI-qRT-F | AAGTGCTCTGGTACGTGCTC | Solyc04g071990.2 |
| SlGI-qRT-R | TCGGCTGTTAGCTTCCCAAG |  |
| SlRVE8-qRT-F | ATGCAAGTGGATGCCTGTCA | Solyc10g005080.2 |
| SlRVE8-qRT-R | GAGCGATCTACCTGCTTCCC |  |
| SlLNK1-qRT-F | TCAGACTACGGTGGCTGGTA | Solyc04g009050.2 |
| SlLNK1-qRT-R | TCCCGTGCTGACACTTTCAA |  |
| SlEID1-qRT-F | AGTTCGACGTCTTCGTGTCC | Solyc09g075080.2 |
| SlEID1-qRT-R | GCCAAATGTGAACAAGGCGA |  |
| SlLHCB1-qRT-F | GAGCTTTTGGCACGTAACGG | Solyc03g005760.1 |
| SlLHCB1-qRT-R | CAAGAGGCCCACCAGCAATA |  |
| SlTUB-qRT-F | CAACCTCTGTGGTGGAACCT | Solyc04g077020.3 |
| SlTUB-qRT-R | TGGTGTATGTGGGACGCTCAA |  |
| SlpsbA-qRT-F | CTAGCTGCTTGGCCTGTAGT | Solyc00g500329.1.1 |
| SlpsbA-qRT-R | CCATACCAAGGTTAGCACGGT |  |
| SlRbcS1-qRT-F | CACCCTTCACCGGACTCAAA | Solyc02g063150.3.1 |
| SlRbcS1-qRT-R | CACCTGCATGCATCTAACGC |  |
| SlatpA-qRT-F | ACAATCCCAATCAGCCCCTC | Solyc00g500050.1.1 |
| SlatpA-qRT-R | GGCTTCTGCTTCCTCGGTAA |  |
